# The m^6^A Readers YTHDF1 and YTHDF2 Synergistically Control Cerebellar Parallel Fiber Growth by Regulating Local Translation of the Key Wnt5a Signaling Components in Axons

**DOI:** 10.1002/advs.202101329

**Published:** 2021-10-12

**Authors:** Jun Yu, Yuanchu She, Lixin Yang, Mengru Zhuang, Peng Han, Jianhui Liu, Xiaoyan Lin, Nijia Wang, Mengxian Chen, Chunxuan Jiang, Yujia Zhang, Yujing Yuan, Sheng‐Jian Ji

**Affiliations:** ^1^ School of Life Sciences Department of Biology Shenzhen Key Laboratory of Gene Regulation and Systems Biology Brain Research Center Southern University of Science and Technology Shenzhen Guangdong 518055 China; ^2^ SUSTech‐HKU Joint PhD Program School of Biomedical Sciences Li Ka Shing Faculty of Medicine The University of Hong Kong Hong Kong China

**Keywords:** cerebellar parallel fibers, local translation, m^6^A, YTHDF1, YTHDF2

## Abstract

Messenger RNA m^6^A modification is shown to regulate local translation in axons. However, how the m^6^A codes in axonal mRNAs are read and decoded by the m^6^A reader proteins is still unknown. Here, it is found that the m^6^A readers YTHDF1 and YTHDF2 are both expressed in cerebellar granule cells (GCs) and their axons. Knockdown (KD) of YTHDF1 or YTHDF2 significantly increases GC axon growth rates in vitro. By integrating anti‐YTHDF1&2 RIP‐Seq with the quantitative proteomic analysis or RNA‐seq after KD of YTHDF1 or YTHDF2, a group of transcripts which may mediate the regulation of GC axon growth by YTHDFs is identified. Among them, *Dvl1* and *Wnt5a*, encoding the key components of Wnt pathway, are further found to be locally translated in axons, which are controlled by YTHDF1 and YTHDF2, respectively. Specific ablation of *Ythdf1* or *Ythdf2* in GCs increases parallel fiber growth, promotes synapse formation in cerebellum in vivo, and improves motor coordination ability. Together, this study identifies a mechanism by which the m^6^A readers YTHDF1 and YTHDF2 work synergistically on the Wnt5a pathway through regulating local translation in GC axons to control cerebellar parallel fiber development.

## Graphical abstract

The m^6^A readers, YTHDF1 and YTHDF2, negatively regulate axon growth of cerebellar granule cells (GCs) by mediating local translation of *Dvl1* and *Wnt5a* in axons, respectively. Conditional knockout (cKO) of *Ythdf1* or *Ythdf2* in cerebellar GCs enhances parallel fiber growth and GC‐PC (Purkinje cells) synapse formation, which eventually improves the motor coordination ability of cKO mice.

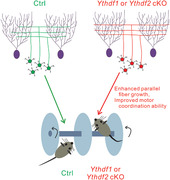

## 1 Introduction

Messenger RNAs can be targeted and locally translated in axons in response to extrinsic cues to regulate axon growth and guidance.^[^ ^1^, ^2^, ^3^, ^4^, ^5^ ^]^ Recent findings suggest that *N* ^6^‐methyladenosine (m^6^A) modification regulates local translation of mRNA in axons.^[^ ^6^ ^]^ However, how these methylated axonal mRNAs are recognized and decoded by their readers in axons is still not known.

Previous studies have suggested that the major m^6^A readers YTHDF1 and YTHDF2 have almost opposite functions in determining the fate of their m^6^A‐modified target transcripts: the former enhancing their translation,^[^ ^7^ ^]^ while the latter destabilizing them.^[^ ^8^ ^]^ Whether and how these apparently counteracting mechanisms interact with each other to regulate biological processes is not known. In addition, recent studies suggest that YTHDFs work redundantly to mediate mRNA degradation.^[^ ^9^, ^10^ ^]^ These debatable models and theories highlight the requirement for further exploration on the functions and mechanisms of the m^6^A readers on their target mRNAs in regulating biological processes.

Wnt family proteins are a group of highly conserved secreted morphogens that play important roles during neuronal development.^[^ ^11^ ^]^ During the early stages of neural circuit formation, Wnt5a signaling has been shown to control neuronal polarity, promote axon growth, and regulate axon guidance.^[^ ^12^, ^13^, ^14^ ^]^ Wnt5a works through a noncanonical Wnt signaling pathway by activating Frizzled3 (Fzd3), which is facilitated by Van Gogh/Strabismus (Vangl2) but antagonized by Dishevelled‐1 (Dvl1).^[^ ^12^ ^]^ Thus, Dvl1 blocks Wnt5a signaling. These previous studies mainly focused on the paracrine Wnt5a signaling. Whether Wnt5a works in an autocrine way and whether Wnt5a signaling is regulated by local translation in axons are still not known.

The cerebellum plays a vital role in controlling motor learning and movement coordination. The two major neuron types in cerebellum are granule cells (GCs) and Purkinje cells (PCs). Postmitotic GCs accumulate in the deeper layer of the external granule layer (EGL) and extend opposing bipolar axons horizontally to the cerebellar folia surface. These nascent parallel fibers will ultimately innervate and form synapses with PCs after GC somata migrate through the molecular layer (ML) to the inner granule layer to finally become mature GCs. However, little is known about the mechanisms regulating parallel fiber growth, which is a key step in cerebellar GC‐PC circuit formation.

Here, we found that the m^6^A readers YTHDF1 and YTHDF2 are highly expressed in GC axons and knockdown (KD) of either YTHDF1 or YTHDF2 in cultured GCs significantly promotes axon growth in vitro. We further demonstrated that YTHDF1 and YTHDF2 synergistically regulate the Wnt5a signaling to control GC axon growth. Both *Wnt5a* and *Dvl1* mRNA are modified by m^6^A and targeted to GC axons, where their local translations are regulated by YTHDF2 and YTHDF1, respectively. Specific ablation of *Ythdf1* or *Ythdf2* from GCs does not affect their neurogenesis. However, both *Ythdf1* and *Ythdf2* cKO mice showed enhanced parallel fiber (PF) length and increased formation of synapses compared to control mice. Interestingly, the motor coordination ability was significantly improved in both *Ythdf1* and *Ythdf2* cKO mice. This study identifies the m^6^A readers YTHDF1 and YTHDF2 as negative regulators for cerebellar PF growth.

## 2 Results

### 2.1 Knockdown of YTHDF1 and YTHDF2 Promoted GC Axon Growth In Vitro

**Figure 1:**
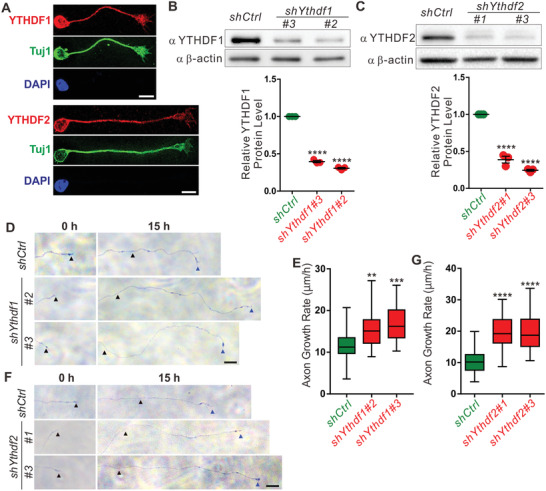
KD of YTHDF1 or YTHDF2 significantly promoted GC axon growth in vitro. A) Representative confocal images showing YTHDF1 and YTHDF2 are expressed in the growth cones and axons of cultured P6–P8 GCs. B) Western blotting (WB) validating the KD efficiency of *shYthdf1* in cultured GCs. Data of quantification are mean ± SEM and represented as dot plots (*n* = 3): *shYthdf1#2* versus *shCtrl*, ****p = 3.91E‐08; *shYthdf1#3* versus *shCtrl*, ****p = 8.62E‐09; by one‐way ANOVA followed by Tukey's multiple comparison test. C) WB validating the KD efficiency of *shYthdf2* in cultured GCs. Data of quantification are mean ± SEM and represented as dot plots (*n* = 3): *shYthdf2#1 *versus *shCtrl*, ****p = 9.15E‐06; *shYthdf2#3* versus *shCtrl*, ****p = 2.53E‐06; by one‐way ANOVA followed by Tukey's multiple comparison test. D) Representative images showing that axon growth rates of GCs are significantly increased after KD of YTHDF1. Black and blue arrowheads indicate the terminals of the same axons imaged at 0 and 15 h, respectively. E) Quantification of axon growth rates in (D). Data are represented as box and whisker plots: *shYthdf1#2* versus *shCtrl*, **p = 0.0030; *shYthdf1#3* versus *shCtrl*, ***p = 1.39E‐04; *n* = 24 axons for each group; by one‐way ANOVA followed by Tukey's multiple comparison test. F) Representative images showing that axon growth rates of GCs are significantly increased after KD of YTHDF2. Black and blue arrowheads indicate the terminals of the same axons imaged at 0 and 15 h, respectively. G) Quantification of axon growth rate in (F). Data are represented as box and whisker plots: *shYthdf2#1 *versus *shCtrl*, ****p = 1.40E‐07; *shYthdf2#3* versus *shCtrl*, ****p = 6.38E‐08; *n* = 24 axons for each group; by one‐way ANOVA followed by Tukey's multiple comparison test. A,D,F) Scale bars represent 10 µm.

In order to investigate the roles of YTHDF1 and YTHDF2 in cerebellar GC axon development, we first checked their expressions in GCs. The cerebella of postnatal day 6–8 (P6–P8) wildtype (WT) mouse pups were dissected, dissociated, and cultured in vitro. Immunostaining of GCs with YTHDF1 and YTHDF2 antibodies showed that both YTHDF1 and YTHDF2 were expressed in GC somata, axons, and growth cones (**Figure** **1A**). We generated lentiviral shRNAs against *Ythdf1* and *Ythdf2*, which showed efficient KD of YTHDF1 and YTHDF2, respectively (Figure 1B,C). Immunofluorescence (IF) signals of both YTHDF1 and YTHDF2 in GC axons were also significantly reduced after infection of *shYthdf1* or *shYthdf2* (Figure S1A–D, Supporting Information), suggesting that axonal YTHDF1 and YTHDF2 signals are specific. Our previous work showed that m^6^A modification of axonal mRNA can regulate axon growth,^[^ ^6^ ^]^ and here we found that both YTHDF1 and YTHDF2 were expressed in GC axons. So we wondered whether YTHDF1 and YTHDF2 are the m^6^A readers to mediate axon growth. GCs from P6–P8 WT mouse cerebella were cultured and infected with *shYthdf1*, *shYthdf2*, or *shCtrl* lentivirus. After puromycin selection, GC axons were imaged at two time points and axon growth rates were measured. KD of YTHDF1 by two different *shYthdf1* significantly increased axon growth rates, compared with *shCtrl* (Figure 1D,E). Interestingly, KD of YTHDF2 by two different *shYthdf2* also significantly increased axon growth rates (Figure 1F,G). Thus, both YTHDF1 and YTHDF2 negatively regulate axon growth of GCs, suggesting that they might work synergistically in this process.

### 2.2 Target mRNAs of YTHDF1 and YTHDF2 were Identified by Integrating Transcriptome, Epitranscriptome, and Proteome Analyses

**Figure 2:**
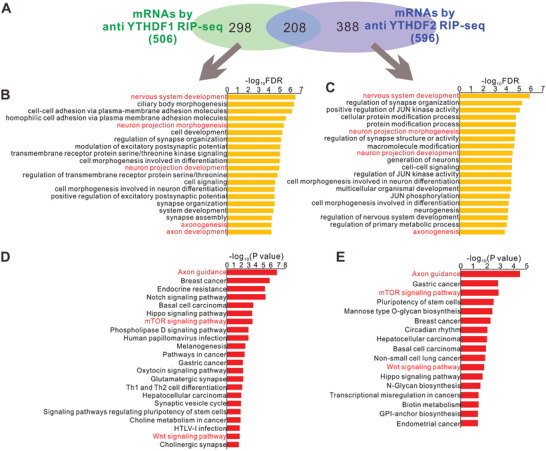
The putative mRNA targets were identified by anti‐YTHDF1 and anti‐YTHDF2 RIP‐seq. A) Venn diagram showing numbers of mRNA targets identified by anti‐YTHDF1 and anti‐YTHDF2 RIP‐seq. B,C) GO analysis of target mRNAs identified by B) anti‐YTHDF1 and C) anti‐YTHDF2 RIP‐seq. The GO terms in Biological Process are shown. The most relevant terms are highlighted in red texts. D,E) KEGG analysis of target mRNAs identified by D) anti‐YTHDF1 and E) anti‐YTHDF2 RIP‐seq. Axon guidance, mTOR signaling pathway, and Wnt signaling pathway are highlighted in red texts.

In order to investigate the mechanisms involved in YTHDF1/YTHDF2‐regulated GC axon growth, we first performed RNA immunoprecipitation‐sequencing (RIP‐seq) to find out the mRNAs that can be recognized and bound by YTHDF1 and YTHDF2 in GCs. By anti‐YTHDF1 and anti‐YTHDF2 RIP‐seq, 506 and 596 mRNAs were pulled down from P6–P8 GCs, respectively (**Figure** **2A** and Tables S1 and S2, Supporting Information). Gene ontology (GO) analysis of those mRNA showed that they were enriched in biological processes such as nervous system development, neuron projection morphogenesis, neuron projection development, axonogenesis, and axon development (Figure 2B,C; also see Figure S2 in the Supporting Information for GO terms in cellular components), which is consistent with the regulation of GC axon growth by YTHDF1 and YTHDF2. KEGG analysis of signaling pathways showed that those mRNAs were enriched in axon guidance, mTOR signaling pathway, and Wnt signaling pathway (Figure 2D,E), further supporting the roles of YTHDF1 and YTHDF2 in controlling GC axon development and regulating translation and the Wnt pathway.

**Figure 3:**
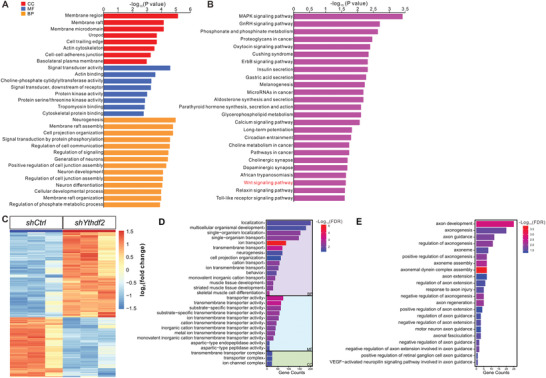
The differentially expressed genes were identified by proteome and transcriptome analysis after KD of YTHDF1 and YTHDF2, respectively. A) GO analysis of downregulated proteins revealed by quantitative proteomic analysis after YTHDF1 KD in GCs. B) KEGG analysis of downregulated proteins revealed by quantitative proteomic analysis. The Wnt signaling pathway is highlighted in red texts. C) Heatmap showing the differential expression profiling of genes by RNA‐seq after YTHDF2 KD in GCs. D) GO analysis of differentially expressed genes revealed by RNA‐seq after YTHDF2 KD in GCs. E) Axon‐related GO terms of differentially expressed genes revealed by RNA‐seq after YTHDF2 KD in GCs. The GO terms in Biological Process are shown. BP, biological process; MF, molecular function; CC, cellular component.

We continued to perform quantitative proteomic analysis after KD of YTHDF1 in GCs using mass spectrometry (MS) which will detect protein level changes for its target mRNAs in either working models for YTHDF1 (regulating translation or stability of its target mRNAs). We discovered 352 proteins that were differentially expressed (change fold >1.2) after YTHDF1 KD (Table S3, Supporting Information). GO analysis of these differentially expressed proteins showed that they were enriched in the cellular components such as synapse, synapse part, and plasma membrane part (Figure S3A, Supporting Information). We further focused on the 142 downregulated proteins and GO analysis showed that they were enriched in the cellular components such as membrane region, membrane raft, and cytoskeleton; the molecular functions such as signal transducer, actin binding, and cytoskeletal protein binding; and the biological functions such as cell projection organization, regulation of signaling and so on (**Figure** **3A**). All these are consistent with the regulation of GC axon growth by YTHDF1. KEGG analysis of downregulated proteins and all the differentially expressed proteins showed that they were noteworthily enriched in the Wnt signaling pathway (Figure 3B and Figure S3B, Supporting Information).

We also performed RNA‐seq after KD of YTHDF2 in GCs since YTHDF2 is widely accepted to regulate the stability of its target mRNAs. Altogether we found 923 mRNA that were differentially regulated after YTHDF2 KD, among which 587 mRNA were upregulated (Figure 3C and Table S4, Supporting Information). GO analysis of all differentially expressed genes showed that they were enriched in localization, neurogenesis, cell projection organization, and so on (Figure 3D). We continued to zoom in to check neural terms and found that many of them were associated with axon development, axonogenesis, axon guidance, and axon extension (Figure 3E), which are consistent with the regulation of GC axon growth by YTHDF2.

Next, we integrated anti‐YTHDF1 RIP‐seq data with YTHDF1‐KD/MS data and anti‐YTHDF2 RIP‐seq data with YTHDF2‐KD/RNA‐seq data to identify the mRNA targets that directly mediate YTHDF1/2‐regulated GC axon growth. As shown in Table S5 in the Supporting Information, we revealed 11 YTHDF1 target mRNAs with their protein levels regulated after YTHDF1 KD, and 11 YTHDF2 target mRNAs with their mRNA levels regulated after YTHDF2 KD. Most of these mRNAs have been found to be modified by m^6^A in mouse cerebellum.^[^ ^15^, ^16^ ^]^

We also performed transcriptome analysis after YTHDF1 KD (Table S6, Supporting Information). Interestingly, the 11 YTHDF1 target mRNAs with their protein levels regulated after YTHDF1 KD did not show mRNA level change from the YTHDF1‐KD/RNA‐seq data (Table S7, Supporting Information), suggesting a mechanism that YTHDF1 regulates the translation, but not the stability of these m^6^A‐modified mRNAs. We also integrated anti‐YTHDF1 RIP‐seq data with YTHDF1‐KD/RNA‐seq data to identify the YTHDF1 targets that were regulated in their mRNA levels. As shown in Table S7 in the Supporting Information, we revealed 12 YTHDF1 target mRNAs with their mRNA levels changed after YTHDF1 KD. However, none of them was detected in the YTHDF1‐KD/MS experiment (Table S7, Supporting Information). All these results suggest that the protein levels of YTHDF1 targets are mainly controlled at the translational level instead of mRNA levels.

In summary, we identified the target mRNAs of YTHDF1 and YTHDF2 in cerebellar development by integrating transcriptome, epitranscriptome, and proteome analyses. The translation or stability of these mRNAs was controlled by YTHDF1 and YTHDF2, respectively (Table S5, Supporting Information).

### 2.3 YTHDF1 and YTHDF2 Regulate Local Translation of *Dvl1* and *Wnt5a*, Respectively, to Control GC Axon Growth

**Figure 4:**
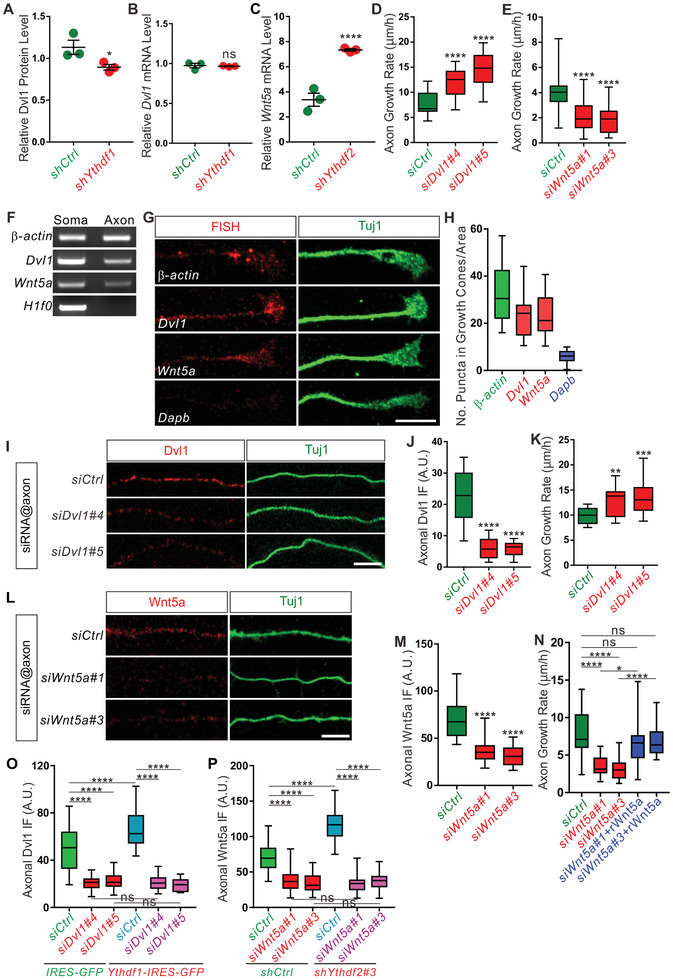
YTHDF1 and YTHDF2 regulate local translation of *Dvl1* and *Wnt5a*, respectively, to control the GC axon growth. A) Relative Dvl1 protein level detected by TMT‐labeled proteomic analysis after YTHDF1 KD. Data are mean ± SEM: *p = 0.047; *n* = 3 replicates; by unpaired Student's *t* test. B) RT‐qPCR confirming the *Dvl1* mRNA level was unchanged after KD of YTHDF1 in GCs. Data are mean ± SEM: *p* = 0.78; *n* = 3; ns, not significant; by unpaired Student's *t* test. C) Relative *Wnt5a* mRNA level measured by RNA‐seq after YTHDF2 KD. Data are mean ± SEM: ****p = 4.92E‐05; *n* = 3 replicates; by unpaired Student's *t* test. D) Axon growth rate significantly increased after KD of Dvl1. Quantification of axon growth rates after KD of Dvl1 using siRNAs. Data are represented as box and whisker plots: *n* = 21 axons for each group; *siDvl1#4 *versus *siCtrl*, ****p = 2.09E‐05; *siDvl1#5 *versus *siCtrl*, ****p = 3.15E‐10. All by one‐way ANOVA followed by Tukey's multiple comparison test. E) Axon growth rate significantly decreased after KD of Wnt5a. Quantification of axon growth rates after KD of Wnt5a using siRNAs. Data are represented as box and whisker plots: *n* = 20 axons for each group; *siWnt5a#1 *versus *siCtrl*, ****p = 4.79E‐05; *siWnt5a#3* versus *siCtrl*, ****p = 4.96E‐06. All by one‐way ANOVA followed by Tukey's multiple comparison test. F) *Dvl1* and *Wnt5a* mRNAs were detected in axons by RT‐PCR using total RNA from pure axons or soma, respectively. Similar to *β‐actin* mRNA which is a positive control for axonal mRNAs, *Dvl1* and *Wnt5a* mRNAs were detected in both axons and soma. The absence of *H1f0* mRNA from axons indicated that the axonal material was pure with no soma incorporation. G,H) Detection of *Dvl1* and *Wnt5a* mRNA localization in growth cones of GC neurons by FISH. Dissociated GCs were cultured for 2 DIV and then FISH was performed using RNAscope riboprobes. *Dvl1* and *Wnt5a* mRNAs were detected in growth cones of GC neurons as red punctate patterns. *β‐actin and Dapb* serve as positive and negative controls, respectively. Tuj1 immunostaining was used to visualize axons. Quantification of puncta density was shown in (H). I,J) Compartmentalized KD of Dvl1 in GC axons. GCs were cultured in microfluidic chambers and *siDvl1* was specifically transfected to axons only. Compared with *siCtrl, siDvl1#4* and *siDvl1#5* led to significant decrease of Dvl1 IF signals. Quantification data are represented as box and whisker plots (J). *siDvl1#4* (*n* = 15 axons) versus *siCtrl* (*n* = 18 axons), ****p = 1.11E‐11; *siDvl1#5* (*n* = 17 axons) versus *siCtrl*, ****p = 3.56E‐12; by one‐way ANOVA followed by Tukey's multiple comparison test. K) Axon growth rates significantly increased after axon‐specific KD of Dvl1. Data are represented as box and whisker plots. *siDvl1#4* (*n* = 18 axons) versus *siCtrl* (*n* = 21 axons), **p = 0.0024; *siDvl1#5* (*n* = 17 axons) versus *siCtrl*, ***p = 0.00049; by one‐way ANOVA followed by Tukey's multiple comparison test. L,M) Compartmentalized KD of Wnt5a in axons. Compared with *siCtrl*, *siWnt5a#1* and *siWnt5a#3* led to significant decrease of Wnt5a IF signals. Quantification data are represented as box and whisker plots (M). *siWnt5a#1* (*n* = 38 axons) versus *siCtrl* (*n* = 32 axons), ****p = 6.80E‐14; *siWnt5a#3* (*n* = 39 axons) versus *siCtrl*, ****p = 5.20E‐14; by one‐way ANOVA followed by Tukey's multiple comparison test. N) Axon growth rates significantly decreased after axon‐specific KD of Wnt5a which can be rescued by application of recombinant Wnt5a protein into axonal compartments. Data are represented as box and whisker plots. *siWnt5a#1* (*n* = 15 axons) versus *siCtrl* (*n* = 16 axons), ****p = 3.86E‐06; *siWnt5a#3* (*n* = 19 axons) versus *siCtrl*, ****p = 1.59E‐07; *siWnt5a#1*+rWnt5a (*n* = 18 axons) versus *siCtrl*, *p* = 0.34; *siWnt5a#3*+rWnt5a (*n* = 16 axons) versus *siCtrl*, *p* = 0.85; *siWnt5a#1*+rWnt5a versus *siWnt5a#1*, *p = 0.036; *siWnt5a#3*+rWnt5a versus *siWnt5a#3*, ****p = 3.40E‐05; ns, not significant; by one‐way ANOVA followed by Tukey's multiple comparison test. O) Overexpression of YTHDF1 increased axonal Dvl1 protein level in cultured GCs and axon‐specific *siDvl1* KD eliminated this increase. Data are represented as box and whisker plots. *Ythdf1‐IRES‐GFP* + *siCtrl* versus *IRES‐GFP* + *siCtrl*, ****p = 1.34E‐05; *Ythdf1‐IRES‐GFP* + *siDvl1#4* versus *IRES‐GFP* + *siDvl1#4*, *p* = 0.99; *Ythdf1‐IRES‐GFP* + *siDvl1#5* versus *IRES‐GFP* + *siDvl1#5*, *p* = 0.84; *IRES‐GFP* + *siDvl1#4* versus *IRES‐GFP* + *siCtrl*, *p* = 5.60E‐14; *IRES‐GFP* + *siDvl1#5* versus *IRES‐GFP* + *siCtrl*, *p* = 5.80E‐14; *Ythdf1‐IRES‐GFP* + *siDvl1#4* versus *Ythdf1‐IRES‐GFP* + *siCtrl*, ****p = 1.01E‐15; *Ythdf1‐IRES‐GFP* + *siDvl1#5* versus *Ythdf1‐IRES‐GFP* + *siCtrl*, ****p = 1.02E‐15; ns, not significant; *n* = 27 axons for each group; by one‐way ANOVA followed by Tukey's multiple comparison test. P) KD of YTHDF2 increased axonal Wnt5a protein level in GCs and axon‐specific *siWnt5a* KD eliminated this increase. Data are represented as box and whisker plots. *shYthdf2#3* + *siCtrl* versus *shCtrl* + *siCtrl*, ****p = 4.30E‐15; *shYthdf2#3* + *siWnt5a#1* versus *shCtrl* + *siWnt5a#1*, *p* = 0.99; *shYthdf2#3* + *siWnt5a#3* versus *shCtrl* + *siWnt5a#3*, *p* = 0.89; *shCtrl* + *siWnt5a#1* versus *shCtrl* + *siCtrl*, ****p = 1.34E‐11; *shCtrl* + *siWnt5a#3* versus *shCtrl* + *siCtrl*, *p* = 1.01E‐13; *shYthdf2#3* + *siWnt5a#1* versus *shYthdf2#3* + *siCtrl*, ****p = 1.01E‐15; *shYthdf2#3* + *siWnt5a#3* versus *shYthdf2#3* + *siCtrl*, ****p = 1.02E‐15; ns, not significant; *n* = 27 axons for each group; by one‐way ANOVA followed by Tukey's multiple comparison test. Scale bars represent G) 10 µm and I,L) 5 µm.

The results of YTHDF1/2 RIPseq, YTHDF1‐KD/MS, and YTHDF2‐KD/RNAseq for identifying YTHDF targets pointed to the Wnt signaling pathway (Figure 2 and 3), and especially its key components Wnt5a and Dvl1 (Table S5, Supporting Information). After YTHDF1 KD, Dvl1 protein level was downregulated by MS analysis, without changing *Dvl1* mRNA level (**Figure** **4A**,B), suggesting a mechanism that YTHDF1 regulates the translation, but not the stability of *Dvl1* mRNA. After YTHDF2 KD, *Wnt5a* mRNA level was upregulated by RNA‐seq analysis (Figure 4C), suggesting a mechanism that YTHDF2 normally destabilizes *Wnt5a* mRNA. Next we checked the functions of those target genes in GC axon growth. Previous studies have shown that Wnt5a stimulated axon growth and regulated axon guidance by activating its receptor Frizzled3, which could be inhibited by Dvl1.^[^ ^12^, ^13^, ^14^ ^]^ We first designed siRNA against each of those mRNAs and validated their KD efficiencies (Figure S4A,B). KD of Dvl1 significantly increased GC axon growth rate (Figure 4D). Considering the fact that YTHDF1 KD caused decreases of translation of *Dvl1* mRNA and promoted axon growth, these data support such a model that YTHDF1 enhances translation of the *Dvl1* mRNA and negatively regulates GC axon growth. For YTHDF2 target, we found that KD of Wnt5a significantly decreased axon growth rate (Figure 4E). Considering the fact that YTHDF2 KD stabilized the *Wnt5a* mRNA and promoted axon growth, these data support such a model that YTHDF2 destabilizes the *Wnt5a* mRNA and negatively regulates GC axon growth.

We further validated the respective regulation of *Dvl1* and *Wnt5a* by YTHDF1 and YTHDF2, respectively. Consistent with the proteomic data, the protein level of Dvl1 was significantly reduced after KD of YTHDF1 in GCs and this downregulation was not affected by treatment with the proteasome inhibitor MG132 (Figure S4C,D, Supporting Information). These data suggest that the decreased protein level of Dvl1 after knocking down YTHDF1 was due to the declined translation of *Dvl1* rather than decreased stability of Dvl1 protein. We further co‐transfected plasmids expressing YTHDF1 and HA‐tagged Dvl1 to HEK293T cells. As shown in Figure S4E–G in the Supporting Information, co‐transfection of YTHDF1 upregulated HA‐Dvl1 protein level, without changing *Dvl1* mRNA level. These data suggest that YTHDF1 normally promotes translation of *Dvl1* in GCs. Consistent with the RNA‐seq data showing increased *Wnt5a* mRNA level after YTHDF2 KD in GCs, the stability of *Wnt5a* mRNA was significantly increased after KD of YTHDF2 in GCs by reverse transcription quantitative real‐time polymerase chain reaction (RT‐qPCR; Figure S4H, Supporting Information), suggesting that YTHDF2 normally destabilizing *Wnt5a* mRNA in GCs. All these data and findings support such a model that YTHDF2 and YTHDF1 work synergistically over Wnt5a pathway to negatively regulate GC axon growth by destabilizing *Wnt5a* mRNA and promoting translation of *Dvl1* mRNA, respectively. As both YTHDF1 and YTHDF2 are expressed in GC axons, we wondered whether this synergistic action of YTHDF1 and YTHDF2 works in axons to regulate local translation of their target mRNAs and control GC axon growth.

To test this, we first explored whether *Dvl1* and *Wnt5a* mRNAs were present in GC axons using different approaches. Pure GC axon materials were collected using microfluidic chambers and axonal RNAs were tested by RT‐PCR as previously reported.^[^ ^6^, ^17^, ^18^ ^]^ As shown in Figure 4F, both *Dvl1* and *Wnt5a* mRNA were detected in GC axons, with *β‐actin* and *H1f0* as positive and negative controls. We also carried out the fluorescence in situ hybridization (FISH) to directly check the presence of *Dvl1* and *Wnt5a* mRNA in GC axons. As shown in Figure 4G,H, riboprobes directed against *Dvl1*, *Wnt5a*, and *β‐actin* gave punctate staining patterns in GC axons, especially in the growth cones (*β‐actin* and *Dapb* as positive and negative control probes, respectively).

Next, we tested whether *Dvl1* and *Wnt5a* were locally translated in GC axons. For this, we performed compartmentalized KD in axons with siRNAs against axonal mRNAs, following the previously published procedures.^[^ ^6^, ^17^ ^]^ Axon‐specific KD using *siDvl1* and *siWnt5a* led to significant decreases of the protein levels of Dvl1 and Wnt5a in GC axons by IF, respectively (Figure 4I,J,L,M), without affecting their levels in GC soma (Figure S4I–L, Supporting Information). These results suggest that *Dvl1* and *Wnt5a* are locally translated. We continued to check the effects of inhibiting local translation of *Dvl1* and *Wnt5a* on GC axon growth. Axon‐specific KD of Dvl1 significantly increased axon growth rate (Figure 4K), indicating that local translation of *Dvl1* represses GC axon growth. Axon‐specific KD of Wnt5a significantly inhibited axon growth (Figure 4N), suggesting that local translation of *Wnt5a* promotes GC axon growth. Wnt5a is a secreted protein and might work as an autocrine signal to regulate axon growth. To test this, we applied the recombinant Wnt5a protein to the GC‐axonal‐Wnt5a‐deficient cultures, which sufficiently rescued axon growth (Figure 4N), supporting a model that axonally derived Wnt5a is secreted by and then works back onto GC axons to promote their growth. Taken together, those results demonstrate that *Dvl1* and *Wnt5a* are locally translated to regulate GC axon growth. Next, we explored the regulation of local translation of *Dvl1 and Wnt5a* by YTHDF1 and YTHDF2 in axons. Overexpression of YTHDF1 and KD of YTHDF2 resulted in increased Dvl1 and Wnt5a protein levels in axons, respectively (Figure 4O,P). These increases could be eliminated by axon‐specific KD of *Dvl1* and *Wnt5a* mRNAs using siRNAs, respectively (Figure 4O,P). All these results suggest that YTHDF1 and YTHDF2 regulate intra‐axonal translation of *Dvl1* and *Wnt5a*, respectively, to control GC axon growth.

### 2.4 Conditional Knockout of *Ythdf1* or *Ythdf2* in GCs Promoted Parallel Fiber Growth In Vivo

**Figure 5:**
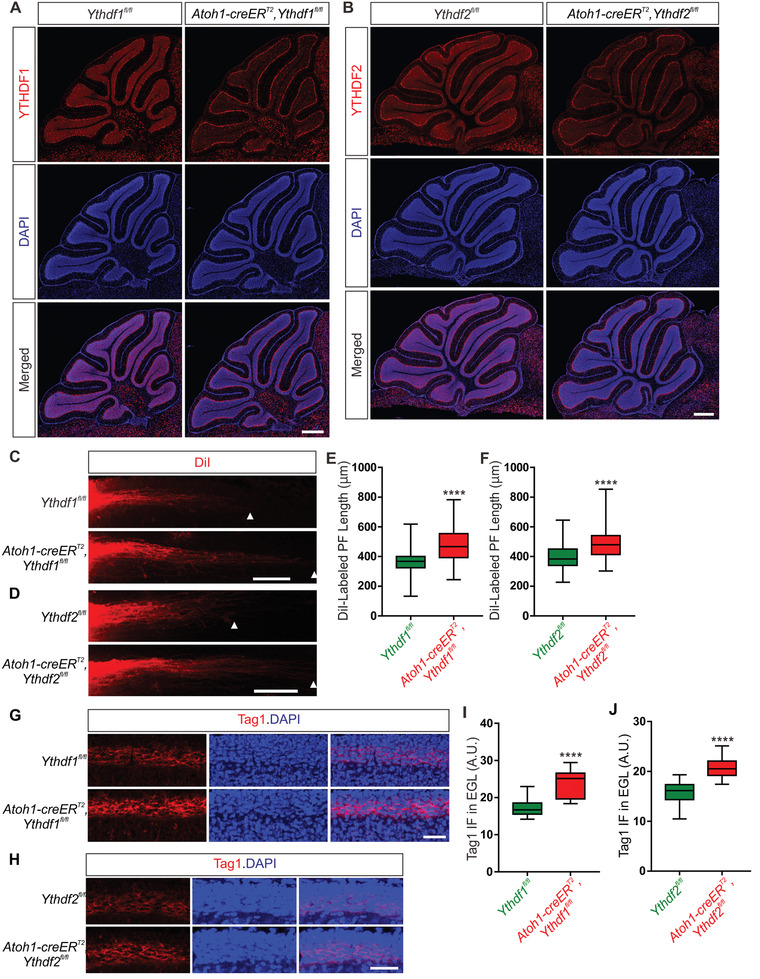
Parallel fiber growth was enhanced in both *Ythdf1* and *Ythdf2* cKO mice. A,B) Representative images of A) YTHDF1 and B) YTHDF2 immunostaining in P15 cerebellum of A) *Ythdf1* and B) *Ythdf2* cKO, respectively. YTHDF1 or YTHDF2 was successfully eliminated in GCs while their expression in PCs was not affected. Scale bars represent 500 µm. C,D) Lengths of parallel fibers labeled by DiI were significantly increased in *Ythdf1* and *Ythdf2* cKO mice. The white arrowheads indicate the terminals of DiI‐labeled PFs. Scale bars represent 100 µm. E,F) Quantification of parallel fiber (PF) lengths in (C) and (D). Data are expressed as box and whisker plots. In (E), ****p = 1.38E‐05; for *Ythdf1^fl/fl^* mice, *n* = 36 confocal fields from 11 pups, for *Ythdf1* cKO mice, *n* = 42 confocal fields from 11 pups. In (F), ****p = 2.29E‐05; for *Ythdf2^fl/fl^* mice, *n* = 46 confocal fields from 13 pups, for *Ythdf2* cKO mice, *n* = 43 confocal fields from 12 pups. All by unpaired Student's *t* test. G,H) Significantly higher Tag1 IF in the deep layer of cerebellar EGL of *Ythdf1* and *Ythdf2* cKO mice was detected. Scale bars represent 40 µm. I,J) Quantification of Tag1 IF intensity signals in (G) and (H). Data are expressed as box and whisker plots. In (I), ****p = 2.80E‐06; *n* = 18 confocal fields for *Ythdf1^fl/fl^* mice, *n* = 15 confocal fields for *Ythdf1* cKO mice. In (J), ****p = 2.02E‐12; *n* = 26 confocal fields for *Ythdf2^fl/fl^* mice, *n* = 28 confocal fields for *Ythdf2* cKO mice. All by unpaired Student's *t* test.

To further explore whether YTHDF1 and YTHDF2 physiologically regulate GC axon growth in vivo, we generated conditional knockout mice by crossing *Ythdf1^fl/fl^* and *Ythdf2^fl/fl^* with *Atoh1‐creER^T2^* mouse line which is predominantly expressed in cerebellar GCs after E12.5 and widely used to generate GC‐specific cKO.^[^ ^19^, ^20^, ^21^, ^22^, ^23^, ^24^ ^]^ YTHDF1 and YTHDF2 were efficiently eliminated from GCs in *Ythdf1* conditional knockout (cKO) and *Ythdf2* cKO (*Ythdf2* cKO) mice at P15, respectively (**Figure** **5A**,B). The expression of YTHDF1 and YTHDF2 in PCs was not affected in either cKO mice (Figure 5A,B). IF of NeuN, a marker for mature cerebellar GCs, showed that conditional knockout of either *Ythdf1* or *Ythdf2* does not disturb neurogenesis of GCs (Figure S5A–D, Supporting Information). To investigate whether the in vitro regulation of GC axon growth by YTHDF1 and YTHDF2 was recapitulated in vivo, we checked parallel fiber development in *Ythdf1* and *Ythdf2* cKO mice by DiI labeling. DiI injected into cerebellar EGL of rodent neonates can label parallel fibers in the deep layer of EGL.^[^ ^25^ ^]^ Compared with control mice, the parallel fibers labeled by DiI in both *Ythdf1* and *Ythdf2* cKO mouse pups at P0 were significantly longer (Figure 5C–F). Direct immunostaining of parallel fibers using an antibody against Tag1, which is a marker for early parallel fibers, showed much higher Tag1 IF intensity in EGL of *Ythdf1* and *Ythdf2* cKO cerebella compared with control mice at P6 (Figure 5G–J). Since KD of YTHDF1 or YTHDF2 did not change Tag1 protein levels in axons (Figure S5E–H, Supporting Information), the increased Tag1 IF intensity in EGL of cKO cerebella indicated the promoted growth of parallel fibers.

In vitro, we found that KD of YTHDF1 inhibited *Dvl1* translation and promoted GC axon growth, while KD of YTHDF2 increased *Wnt5a* mRNA stability and also promoted GC axon growth. So we continued to explore whether the enhanced PF growth in vivo was also caused by these mechanisms. In line with the in vitro results, Dvl1 protein level was significantly decreased in *Ythdf1* cKO mice compared with control (Figure S6A–C, Supporting Information), while *Dvl1* mRNA level was not affected (Figure S6F, Supporting Information). Both *Wnt5a* mRNA and protein levels were significantly increased in *Ythdf2* cKO mice compared with control (Figure S6G–I,L, Supporting Information). The axon markers Tau1 and Tag1 showed increased levels in the cerebellum of *Ythdf1* and *Ythdf2* cKO mice at P15 compared with control mice (Figure S6A,D,E,G,J,K, Supporting Information). These data suggest that YTHDF1 and YTHDF2 regulate translation of *Dvl1* and stability of *Wnt5a*, respectively, and their synergistic action on Wnt5a pathway controls parallel fiber growth in cerebellum.

**Figure 6:**
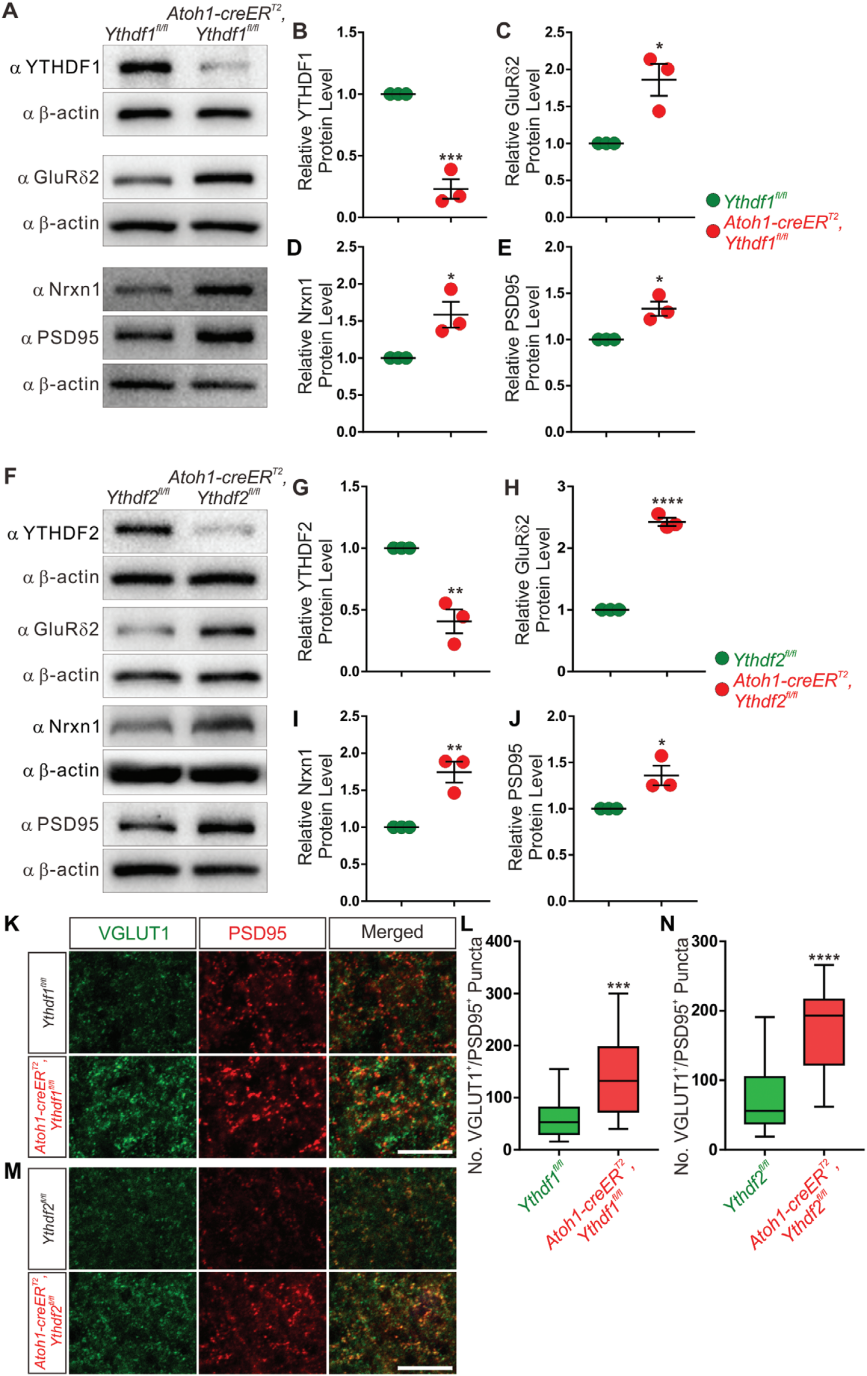
Synapse formation was promoted in *Ythdf1* and *Ythdf2* cKO cerebella. A–E) Representative immunoblots showing that the protein levels of synaptic markers GluR*δ*2, Nrxn1, and PSD95 were increased in A) *Ythdf1* cKO cerebellum at P30. Quantification of B) YTHDF1, C) GluR*δ*2, D) Nrxn1, and E) PSD95. For (B), ***p = 0.00064; for (C), *p = 0.016; for (D), *p = 0.028; for (E), *p = 0.013; *n* = 3 replicates; by unpaired Student's *t* test. F–J) Representative immunoblots showing that GluR*δ*2, Nrxn1, and PSD95 protein levels were increased in F) *Ythdf2* cKO cerebellum at P30. Quantification of G) YTHDF2, H) GluR*δ*2, I) Nrxn1, and J) PSD95. For (G), **p = 0.0037; for (H), ****p = 2.65E‐05; for (I), **p = 0.0062; for (J), *p = 0.028; *n* = 3 replicates; by unpaired Student's *t* test. K–N) Representative images of VGLUT1 and PSD95 co‐immunostaining in the ML of P30 cerebellum of K) *Ythdf1* and M) *Ythdf2* cKO. VGLUT1^+^/PSD95^+^ puncta were counted to measure the number of synapses and quantifications were shown in (L) and (N). Data are expressed as box and whisker plots. In (L), ***p = 1.10E‐04; in (N), ****p = 2.16E‐07; *n* = 20 confocal fields for each group; by unpaired Student's *t* test. Scale bars represent K,M) 5 µm.

As knockout of *Ythdf1* or *Ythdf2* significantly promotes parallel fiber growth, we wondered whether this would enhance synapse formation between parallel fibers and PCs in these cKO mice. The formation of synapses between the parallel fibers of GCs and the dendrites of PCs is mediated by the trans‐synaptic neurexin‐Cbln1‐GluR*δ*2 triad.^[^ ^26^ ^]^ The presynaptic neurexin in PF terminals interacts with postsynaptic glutamate receptor delta2 (GulR*δ*2) through cerebellin‐1 precursor protein (Cbln1) which is secreted by GCs.^[^ ^27^ ^]^ GluR*δ*2 is selectively expressed in PCs and exclusively localized in parallel fiber‐PC synapses.^[^ ^28^, ^29^ ^]^ So we checked the protein levels of GluR*δ*2 and neurexin1 (Nrxn1) in P30 cerebellum by WB. Both GluR*δ*2 and Nrxn1 protein levels were significantly increased in *Ythdf1* and *Ythdf2* cKO mice compared with control mice (**Figure** **6A**–D,F–I). We also checked PSD95, a general postsynaptic marker. PSD95 protein level was also increased in *Ythdf1* and *Ythdf2* cKO cerebella (Figure 6A,E,F,J). We further measured the synapse numbers in vivo by co‐immunostaining of the presynaptic marker VGLUT1 and the postsynaptic marker PSD95. As shown in Figure 6K–N, quantification of VGLUT1^+^/PSD95^+^ puncta indicated that the synapse numbers in the ML of P30 *Ythdf1* and *Ythdf2* cKO cerebella increased compared with their control, respectively. Taken together, these results suggest that due to enhanced parallel fiber growth, the PF→PC synapse formation is promoted in *Ythdf1* and *Ythdf2* cKO mice.

### 2.5 Motor Coordination Ability is Improved in *Ythdf1* and *Ythdf2* cKO Mice

**Figure 7:**
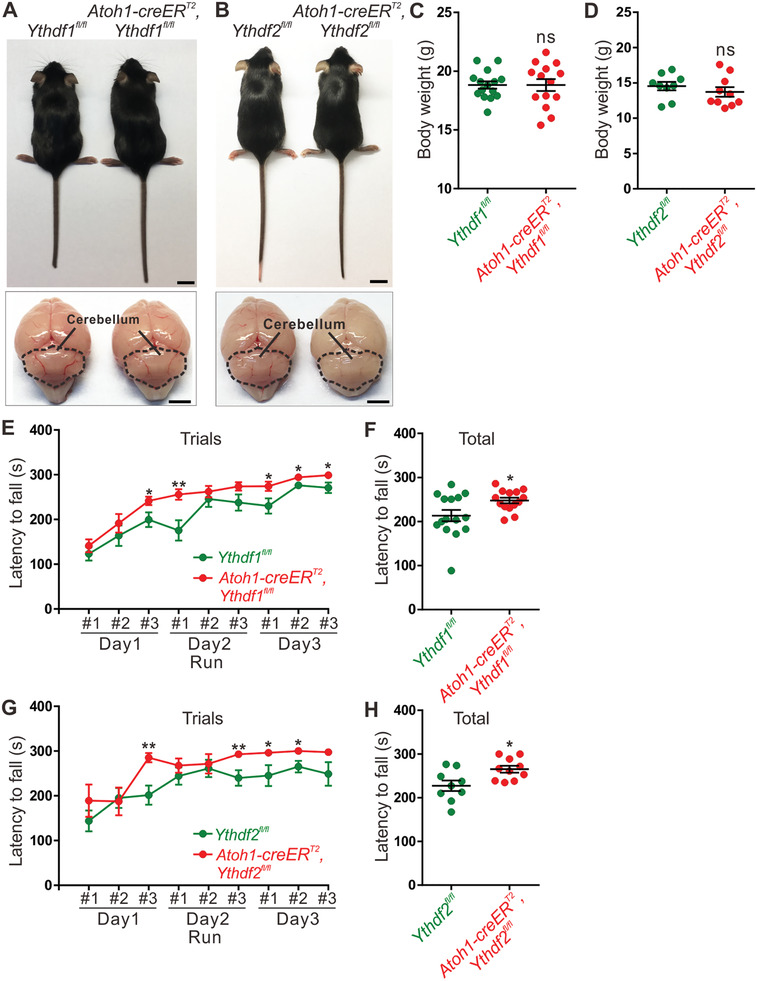
The motor coordination ability is enhanced in *Ythdf1* and *Ythdf2* cKO mice. A) *Ythdf1* and B) *Ythdf2* cKO showed normal animal size and cerebellar development at P40. Scale bars in the upper panels represent 1 cm and scale bars in the lower panels represent 0.25 cm. C,D) Normal body weight in *Ythdf1* and *Ythdf2* cKO mice. In (C), *p* = 0.99; *n* = 15 for *Ythdf1^fl/fl^* mice; *n* = 14 for *Ythdf1* cKO mice. In (D), *p* = 0.37; *n* = 9 for *Ythdf2^fl/fl^* mice; *n* = 10 for *Ythdf2* cKO mice; ns, not significant. All by unpaired Student's *t* test. E,F) The latency to fall measurements for E) each and F) total trial in rotarod test of *Ythdf1* cKO mice. In (E), for Day1‐Run #3, *p = 0.037; for Day2‐Run #1, **p = 0.0049; for Day3‐Run #1, *p = 0.039; for Day3‐Run #2, *p = 0.034; for Day3‐Run #3, *p = 0.037. In (F), *p = 0.027; *n* = 15 for *Ythdf1^fl/fl^* mice; *n* = 14 for *Ythdf1* cKO mice. All by unpaired Student's *t* test. G,H) The latency to fall measurements for G) each and H) total trial in rotarod test of *Ythdf2* cKO mice. In (G), for Day1‐Run #3, **p = 0.0020; for Day2‐Run #3, **p = 0.0089; for Day3‐Run #1, *p = 0.038; for Day3‐Run #2, *p = 0.013. In (H), *p = 0.015; *n* = 15 for *Ythdf2^fl/fl^* mice; *n* = 14 for *Ythdf2* cKO mice. All by unpaired Student's *t* test.

With increased parallel fiber length and promoted synapse formation in *Ythdf1* and *Ythdf2* cKO mice, we wondered whether those phenotypes would affect its motor coordination ability, which is the most important function that cerebellum serves. Compared with control mice, either *Ythdf1* or *Ythdf2* cKO mice showed no obvious difference in animal size, cerebellar size, or body weight (**Figure** **7A**–D and Figure S7A,B, Supporting Information). Then we carried out a series of motor behavior tests to evaluate their motor abilities. The grip strength measurement in the forelimbs of either *Ythdf1* or *Ythdf2* cKO mice did not show any significant differences compared with their corresponding control mice (Figure S7C,D, Supporting Information). We further assessed the motor coordination and balance of each cKO with an accelerating rotarod. Surprisingly, we found that both *Ythdf1* and *Ythdf2* cKO mice performed significantly better than their controls by measuring the latency to fall in the test (Figure 7E–H), indicating that both *Ythdf1* and *Ythdf2* cKO mice have better motor coordination ability than their control mice. We also evaluated gait by the footprint test in these mice. As shown in Figure S7E,G in the Supporting Information, mice from all groups walked in a straight line with evenly alternating gait. *Ythdf1* cKO mice displayed longer distance of stride and distance of stance‐L compared to control mice, while *Ythdf2* cKO mice exhibited increased distance of sway and distance of stance‐R compared with control mice (Figure S7F,H, Supporting Information). Altogether, those results indicate that knockout of *Ythdf1* or *Ythdf2* in cerebellar GCs can improve the motor coordination ability of mice, suggesting that YTHDF1 and YTHDF2 serve as negative regulators for PF growth and cerebellar functions.

## 3 Discussion

**Figure 8:**
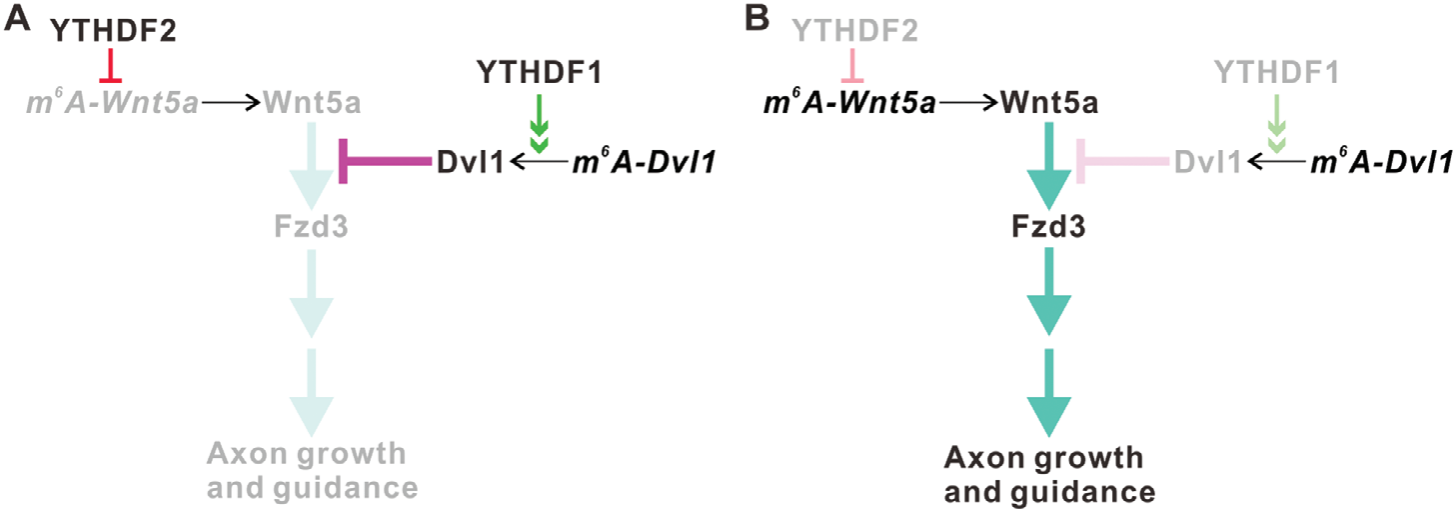
A working model shows that YTHDF1 and YTHDF2 work synergistically to regulate Wnt5a‐PCP signaling pathway and cerebellar granule cell axon growth. A) Under normal conditions, YTHDF1 promotes the translation of m^6^A‐modified *Dvl1* mRNA in GC axons. Dvl1 can block Wnt5a‐Fzd3‐activated PCP signaling. Meanwhile, YTHDF2 facilitates *Wnt5a* mRNA degradation to downregulate Wnt5a protein level in GC axons. So both YTHDF1 and YTHDF2 negatively regulate Wnt5a‐PCP signaling pathway and GC axon growth. B) In *Ythdf1* and *Ythdf2* cKO mice, local translation of *Dvl1* mRNA and decay of *Wnt5a* mRNA in GC axons are inhibited, respectively. The resulting downregulation of Dvl1 and upregulation of Wnt5a protein levels in axons potentiate Wnt5a‐PCP signaling and promote GC axon growth.

m^6^A modification has been shown to regulate axon growth and guidance.^[^ ^6^, ^30^ ^]^ We found that YTHDF1 regulates commissural axon guidance by controlling translation of the axon guidance molecule Robo3.1.^[^ ^30^ ^]^ However, how the m^6^A readers mediate axon growth remains unclear. Here, our study reveals a critical role for the m^6^A readers YTHDF1 and YTHDF2 in cerebellar GC axon growth. YTHDF2 and YTHDF1 work synergistically to regulate Wnt5a pathway by regulating intra‐axonal translation of *Wnt5a* and *Dvl1*, respectively (**Figure** **8**). YTHDF2 and YTHDF1 normally are negative regulators for cerebellar parallel fiber growth. Knockout of *Ythdf1* or *Ythdf2* in cerebellar granule cells promotes GC axon growth by activating Wnt5a‐Frizzled3 pathway (Figure 8 and Figure S8, Supporting Information). The enhanced PF growth promotes synapse formation in cerebellum and improves motor coordination ability in *Ythdf1* and *Ythdf2* cKO mice.

Wnt5a can stimulate axon outgrowth of spinal cord commissural axons through planar cell polarity (PCP) signaling.^[^ ^12^ ^]^ Wnt5a is expressed by the ventral midline cells in the floor plate (FP) with an anterior–posterior gradient which can attract post‐crossing commissural axons to turn anteriorly toward the brain.^[^ ^12^ ^]^ Thus, Wnt5a from FP regulates growth and guidance of commissural axons in a paracrine manner. In this pathway, Wnt5a increases endocytosis of its receptor Frizzled3 in filopodia tips.^[^ ^31^ ^]^ Dvl1 can induce Frizzled3 hyperphosphorylation and accumulation on the plasma membrane, thus blocking the PCP signaling. In this study, we found that Wnt5a signaling pathway is involved in regulating cerebellar GC axon growth. The two key players of Wnt5a signaling pathway, Wnt5a and Dvl1, are locally translated in axons, which is regulated by YTHDF2 and YTHDF1, respectively. Axonally derived Wnt5a is secreted from GC axons. This autonomously secreted Wnt5a works back to regulate GC axon growth in an autocrine manner. Therefore, this study presents a mechanism by which YTHDF1 and YTHDF2 work synergistically to control GC axon growth through regulating Wnt5a‐PCP pathway.

The Dishevelled‐mediated Wnt/PCP signaling has been shown to regulate vertebrate early development such as tissue patterning, morphogenesis, and cell migration, and also has an emerging role in disorders such as cancer progression.^[^ ^32^, ^33^ ^]^ Thus regulation of Wnt5a‐PCP pathway by the synergistic action of YTHDF1 and YTHDF2 may have a broader implication in physiological and pathological conditions.

The precise control of PF length is achieved by balanced actions of positive and negative regulators. The positive regulators include the mouse serine/threonine kinase homologous to *C. elegans* UNC51,^[^ ^34^ ^]^ Discoidin domain receptor 1 (DDR1),^[^ ^35^ ^]^ and transcript factor Pax6.^[^ ^25^ ^]^ The negative regulators include the small GTPase Rho and the Rho‐associated serine/threonine kinase ROCK.^[^ ^36^ ^]^ Here, we demonstrated that the m^6^A readers YTHDF1 and YTHDF2 are two new negative regulators for PF growth by posttranscriptionally regulating local translation of their target mRNAs in axons. Furthermore, the motor coordination ability of *Ythdf1* and *Ythdf2* cKO mice was significantly improved. These findings suggest that these negative regulators might be useful targets to develop drugs or strategies for patients with cerebellar ataxia.

Previous studies have shown that disrupting m^6^A modification in mouse cerebellum by manipulating the m^6^A writer gene *Mettl3* can cause severe developmental defects, including dramatically reduced GC numbers, altered PC patterning, and Bergmann glia architecture.^[^ ^15^, ^16^ ^]^ However, GC‐specific cKO of the two m^6^A reader genes *Ythdf1* or *Ythdf2* does not recapitulate these phenotypes. Indeed, we found that the neurogenesis of GCs or PCs is not affected in these cKO mice. This difference may be due to the fact that the previous studies manipulated *Mettl3* which universally changed the m^6^A landscape, and their manipulations (using Nes‐cre) were in earlier developmental stages and affected the whole cerebellum but not just in GCs. Nevertheless, we found that YTHDF1 and YTHDF2 in GCs do not mediate neurogenesis, which makes it possible to explore their roles in other developmental processes after neurons are born, such as axon growth. In summary, our study identifies a new working mode of m^6^A modification which is mediated by the synergistic actions of the two m^6^A readers YTHDF1 and YTHDF2 on Wnt/PCP pathway to restrict GC axon growth.

## 4 Experimental Section

### Animals and Generation of cKO Mice

For generation of the *Ythdf2^fl/fl^* mice, exon 4 of mouse *Ythdf2* gene was targeted with the consideration that exon 4 encodes the YTH domain and the procedures were the same as the previously reported *Ythdf1 ^fl/fl^* mice.^[^ ^30^ ^]^ *Atoh1‐creER^T2^* mice^[^ ^19^ ^]^ from Jackson Laboratory were used to generate cKO mice. Tamoxifen (13258, Cayman) was dissolved in corn oil at a concentration of 50 mg mL^−1^. 10 mg of Tamoxifen was administrated to E16.5 pregnant mice (midday of the observed plug was considered as E0.5) by intragastric injection. Genotyping primers were as following: *Atoh1‐cre* site: 5’‐TGCCACGCACAAGTGACAGCAATG‐3’ and 5’‐ACCAGAGACGGAAATCCATCGCTC‐3’. The primers for *Ythdf1‐loxP* sites were the same as previously reported.^[^ ^30^ ^]^ The primers for *Ythdf2‐loxP* sites were: the first *Ythdf2‐loxP* site, 5’‐GCTTGTAGTTATGTTGTGTACCAC‐3’ and 5’‐GCAGCTCTGACTATTCTAAAACCTCC‐3’; the second *Ythdf2‐loxP* site, 5’‐CTCATAACATCCATAGCCACAGG‐3’ and 5’‐CCAAGAGATAGCTTTCCTAATG‐3’. All experiments using mice were carried out following animal protocols approved by the Laboratory Animal Welfare and Ethics Committee of Southern University of Science and Technology.

### Cerebellar Granule Cell Culture

Cerebella were dissected out from P6–P8 mice, and meninges were removed in ice‐chilled Hanks' balanced salt solution (HBSS, 14175103, Invitrogen). Then GCs were dissociated in HBSS containing 1% Trypsin (59427C, Sigma) and 0.1% DNase I (DN25, Sigma) at 37 °C with constant agitation. Dissociated GCs were collected and plated in microfluidic chambers or 24‐well plates with coverslips precoated with PDL (100 µg mL^−1^, 3439‐100‐01, Trevigen) and Laminin (3.3 µg mL^−1^, 3400‐010‐02, Trevigen). GCs were cultured in Neurobasal A‐medium (10888‐022, Gibco) with B27 supplement (1x, 17504044, Gibco), GlutaMAX‐1 (1x, 35050–061, Gibco), and penicillin‐streptomycin (1x, 15140‐122, Gibco). For the protein stability assay and the mRNA half‐life assay, MG132 (S2619, Selleck) and actinomycin D (A4448, APEE‐BIO) were used with concentrations of 10 × 10^−6^ and 5 × 10^−6^ m in the neuron culture, respectively.

### Knockdown using Lentiviral shRNA or siRNA and Overexpression using Lentiviral System

The lentiviral plasmids and the lentivirus preparation procedure were described previously.^[^ ^6^, ^30^ ^]^ The target sequences of shRNA were as following: shYthdf1#2: 5′‐GGACATTGGTACTTGGGATAA‐3′; shYthdf1#3: 5′‐GCACACAACCTCTATCTTTGA‐3′

shYthdf2#1: 5′‐GCTCCAGGCATGAATACTATA‐3′; shYthdf2#3: 5’‐GGACGTTCCCAATAGCCAACT‐3’

shCtrl: 5′‐GCATAAACCCGCCACTCATCT‐3′. To select positively infected neurons, puromycin (1 ug mL^−1^, A1113803, Thermo) was added at 2 days postinfection and worked for 24 h. Then experiments of axon growth rate measurement, RNA or protein extraction, and IF were performed. The siRNA‐mediated KD assay was carried out by using GeneSilencer siRNA Transfection Reagent (Genlantis) following the manufacture's manual. The sequences of siRNA were as following: *siDvl1#4*: 5’‐ CCAGUAGCCGGGACGGAAUTT‐3’; *siDvl#5*: 5’‐ GCUUGAAUCUAGCAGCUUUTT‐3’; *siWnt5a#1*: 5’‐GCUGCUAUGUCAAAUGCAATT ‐3’; *siWnt5a#3*: 5’‐GGUGGUCUCUAGGUAUGAATT‐3’; *siCtrl* (RNAi negative control): 5’‐ UUCUCCGAACGUGUCACGUTT‐3’. Experiments such as axon growth rate measurement, RNA or protein extraction, and IF were performed at least 48 h posttransfection of siRNA.

### Plasmid Construction and Cell Assay

The coding sequence (CDS) of *Dvl1* was amplified from whole brain cDNA of P56 mouse by PCR with following primers: 5’‐TACGCTGGCCGGCCAGAATTCATGGCGGAGACCAAAATCATCTACC‐3’ and 5’‐CACTATAGTTCTAGAGGCGCGCCCCACCTTGGCCTGACAGGTGA‐3’. *pCS2‐HA‐Dvl1* was constructed with an expression vector reported previously.^[^ ^30^ ^]^ The *pCAGGS‐Ythdf1‐IRES‐GFP* construct and the assay using the HEK293T cell were reported previously.^[^ ^30^ ^]^

### Axon Growth Assay

To measure axon growth rates, microfluidic chambers were used to culture GCs. Lentiviral infection and siRNA transfection were performed after cells were attached to the coverslip in the soma compartment. Bright‐field images of axons were taken 72 h post shRNA infection or siRNA transfection at different timepoints. Then axon length was manually traced and measured using Image‐Pro Plus software. For axon‐specific KD, *siDvl1* or *siWnt5a* were specifically transfected in axon compartment when axons grew to appropriate lengths.

### Real‐Time RT‐PCR

Total RNA from cultured GCs or cerebella tissue was extracted using Trizol (15596018, Life) reagent. cDNA was synthesized by PrimeScript RT Master Mix (RR036A, Takara) and used for qPCR by SYBR Premix Ex Taq GC (RR071B, Takara). Primers used for qPCR were as following: *Ythdf1: 5’*‐GGGGACAAGTGGTTCTCAGG‐3’ and 5’‐TCCCCAATCTTCAGGCCAAC‐3’, *Ythdf2: 5’*‐ ACAGGCAAGGCCGAATAATG‐3’ and 5’‐GGCTGTGTCACCTCCAGTAG‐3’, *Dvl1: 5’*‐ ATGGCGGAGACCAAAATCATC‐3’ and 5’‐ AACTTGGCATTGTCATCGAAGA‐3’, *Wnt5a:5’*‐ ATGCAGTACATTGGAGAAGGTG‐3’ and 5’‐CGTCTCTCGGCTGCCTATTT‐3’, *GAPDH*: *5’*‐AGGTCGGTGTGAACGGATTTG‐3’ and 5’‐TGTAGACCATGTAGTTGAGGTCA‐3’.

### Western Blotting (WB)

Cultured GCs or cerebellum tissues were homogenized in radioimmunoprecipitation assay buffer (P0013, Beyotime) containing protease inhibitors (4693116001, Roche). Protein was collected by centrifugation and the concentration was measured using the BCA Protein Assay Kit (23227, Thermo). A total of 30 µg boiled protein in each sample was resolved on sodium dodecyl sulfate–polyacrylamide gel electrophoresis gels, then transferred onto polyvinylidene fluoride membranes (ISEQ00010, Millipore). The membranes were then blocked with 5% skim milk or 3% bovine serum albumin (BSA) and incubated with diluted primary antibody at 4 °C overnight. After washing, the membranes were further incubated with diluted secondary antibody conjugated to Horseradish peroxidase (HRP) for 1 h at room temperature (RT). Protein bands were visualized using SuperSignal West Pico PLUS Chemiluminescent Substrate (34580, Thermo) or SuperSignal West Femto Maximum Sensitivity Substrate (34096, Thermo).

Sources and dilutions of antibodies used in WB are as follows: YTHDF1 (17479‐1‐AP, Proteintech) 1:2500; YTHDF2 (24744‐1‐AP, Proteintech) 1:2500; Dvl1 (27384‐1‐AP, Proteintech) 1:1000; Wnt5a (55184‐1‐AP, Proteintech) 1:1000; HA (ab18181, Abcam) 1:2000; Tau1 (MAB3420, Millipore) 1:1000; Tag1 (AF1714, R&D Systems) 1:2500; GluR*δ*2(sc‐26118, Santa Cruz Biotechnology) 1:1000; Nrxn1 (ab77596, Abcam) 1:1000; GAPDH (10494‐1‐AP, Proteintech) 1:1000; *β*‐actin (ab6276, Abcam) 1:30 000; *β*‐actin (AC004, Abclonal) 1:30 000; Donkey anti‐goat IgG H&L (HRP) (ab97110, Abcam) 1:2500; Donkey anti‐mouse IgG H&L (HRP) (ab97030, Abcam) 1:2500; Donkey anti‐rabbit IgG H&L (HRP) (ab16284, Abcam) 1:2500; VHH anti‐mouse IgG secondary antibody (HRP) (KTSM1321, AlpaLife) 1:5000; VHH anti‐rabbit IgG secondary antibody (HRP) (KTSM1322, AlpaLife) 1:5000.

### IF and Immunostaining

P15 or P30 cerebella were freshly embedded in O.C.T and frozen immediately in dry ice. Tissues were then sagittally cryosectioned at 12 µm. For IF of YTHDF1, YTHDF2, or NeuN, the sections were fixed in 4% paraformaldehyde (PFA) for 10 min (YTHDF1 or YTHDF2) or 2 h (NeuN) followed by incubation with blocking solution (5% BSA, 10% donkey serum, 0.25% Triton x‐100) for 1 h at RT. For co‐staining of VGLUT1 and PSD95, a protocol was used which was published previously.^[^ ^37^ ^]^ Briefly, the sections were fixed for 30 s in cold (−20 °C) methanol, rinsed with phosphate‐buffered saline (PBS), permeabilized for 20 min in 0.2% Triton x‐100 in PBS, and blocked for 4 h with 10% donkey serum (SL050, Solarbio) in PBS at RT. Then sections were incubated with primary antibodies diluted in blocking solution overnight at 4 °C: YTHDF1 (1:500), YTHDF2 (1:500), NeuN (1:500, 24307, Cell Signaling Technology), VGLUT1 (1:10 000, 135303, Synaptic Systems), PSD95 (1:100, ab2723, Abcam). After three times wash with PBS, sections were incubated with secondary antibodies diluted in secondary Ab solution (5% BSA, 0.25% Triton x‐100). Finally, slides were mounted with VECTASHIELD Antifade Mounting Medium with 4′,6‐diamidino‐2‐phenylindole (H‐1200, Vector Laboratory).

For Tag1 IF in cerebellar tissues, P6 mice were perfused with PBS followed by 4% PFA. Cerebella were collected and fixed in 4% PFA at 4 °C overnight. Then tissues were dehydrated in 30% sucrose solution for 2 days. After embedded in O.C.T., cerebella were coronally cryosectioned at 12 µm. After antigen retrieval at 93 °C in sodium citrate buffer (pH 6.0), slides were continued to incubate with goat anti‐Tag1 (1:500, AF1714, R&D Systems) in blocking solution at 4 °C overnight. Then after washing with PBS, slides were incubated with Alexa 555 donkey anti‐goat (1:1000, A21432, Thermo) for 1 h at RT before mounting for confocal imaging.

For IF in cultured neurons, cells were rinsed with PBS twice and fixed for 10 min with 4% PFA in 0.1 m PB at RT, then washed with PBS for three times and blocked in PBST (PBS with 1% BSA and 0.1% Triton x‐100; no Triton x‐100 for Fzd3 IF) for 20 min at RT. Antibody incubation conditions were the same as tissue sections and Fzd3 antibody was 1:100 (AF1001, R&D Systems).

All images were captured on Nikon A1R confocal microscope or Zeiss LSM 800 confocal microscope with identical settings for each group in the same experiment. IF intensity was measured using ImageJ software with background intensity subtracted.

### RNA Immunoprecipitation (RIP) and Sequencing (RIP‐Seq)

To perform RIP experiment, the manual of the EZ‐Magna RIP RNA‐Binding Protein Immunoprecipitation Kit (17‐701, Millipore) with minor modifications was followed. Briefly, 1 × 10^7^ granule cells were lysed and then incubated with magnetic beads precoated with 5 µg YTHDF1 antibody (Proteintech, 17479‐1‐AP) or YTHDF2 antibody (Proteintech, 24744‐1‐AP) overnight at 4 °C. RNA was purified using TRIzol reagent. After quality control monitoring using Agilent 2100, 100 ng RNA of input and elutes after RIP was used to generate the library using the TruSeq Stranded RNA Sample Preparation Kit (Illumina) and sequenced on the Illumina HiSeq 3000 platform (Jingneng, Shanghai, China). The filtered reads were mapped to the mouse reference genome (GRCm38) using STAR v2.5 and Cufflinks (version 2.2.1) with default parameters.^[^ ^38^, ^39^ ^]^ Filtered reads were normalized to calculate FPKM (fragments per kilobase of exon per million mapped fragments). To determine which gene is enriched, the FPKM from RIP elute to input was computed and any fold change greater than 2 was considered to be enriched. All enriched genes were used to do the GO analysis. GO enrichment analysis was implemented by the GOseq R package, in which gene length bias was corrected. GO terms with corrected *p* value less than 0.05 were considered significantly enriched.

### RNA‐Seq and Data Analysis

Cultured GCs were infected with lentiviral shRNAs. After puromycin selection, neurons were lysed with Trizol reagent, and total RNA was extracted. A total of 1 µg RNA in each sample was used to generate sequencing libraries using NEBNext Ultra RNA Library Prep Kit for Illumina (NEB) following the manufacturer's protocol and sent for sequencing (Novogene, Beijing, China). Data analysis was performed using the clean data (reads) with high quality, in which low quality reads and reads possessing adapter or ploy‐N were filtered out. The filtered reads were then mapped to the mouse reference genome (GRCm38) using HISAT v2.0.4 and TopHat2 (version 2.1.1) with default parameters.^[^ ^39^, ^40^ ^]^ The numbers of reads mapped to each gene were counted using HTSeq v0.9.1 and the FPKM of each gene was then calculated. The DESeq R package (version 1.18.0) was used to analyze the differentially expressed genes between *shYthdf* vand *shCtrl* (three biological replicates per group). The resulting *p* values were adjusted using the Benjamini and Hochberg's approach for controlling the false discovery rate (FDR). Genes with *p* < 0.05 were considered as differentially expressed and used for GO analysis. GO enrichment analysis was performed through the GOseq R package, in which gene length bias was corrected. GO terms with corrected *p* < 0.05 were considered significantly enriched.

### Quantitative MS

Cultured GCs were infected with lentiviral *shYthdf1* or *shCtrl*. After puromycin selection, neurons were rinsed three times with ice‐cold PBS and then lysed with freshly made lysis buffer (8 m urea (Sigma), 0.1 m HEPES (pH 7.4, Invitrogen)) supplemented with protease inhibitors. Cell lysates were then ultrasonicated on ice and centrifuged at 10 000 × *g* for 10 min at 4 °C. A total of 100 µg protein for each condition was reduced with 5 × 10^−3^ m dithiothreitol (Sigma) for 30 min at 56 °C, followed by alkylation with 11 × 10^−3^ m iodoacetamide (Sigma) for 15 min at RT in the dark. Then the urea concentration in each sample was diluted to less than 2 m by adding 100 × 10^−3^ m triethylammonium bicarbonate (Sigma). Subsequently, protein samples were digested by trypsin (Promega) overnight at 37 °C. Peptides were further desalted by Strata X C18 SPE columns (Phenomenex) and labeled with TMT10plex Mass Tag Labeling kit (Thermo) following the manufacturer's instructions. Finally, the labeled peptides were subjected to high‐performance liquid chromatography fractionation and LC‐MS/MS (liquid chromatography with tandem mass spectrometry) analysis.

### Fluorescence In Situ Hybridization (FISH)

For FISH, cerebellar granule cells were cultured in microfluidic chambers. FISH was carried out using RNAscope Multiplex Fluorescent Reagent Kit with target‐specific double Z probes (Advanced Cell Diagnostics) following the manufacture's protocol. The probe information is as following: *Wnt5a*, target region of 200–1431 (20 pairs); *Dvl1*, target region of 666–2055 (20 pairs); *β‐actin*, target region of 11–869 (15 pairs); *DapB*, target region of 414–862 (10 pairs).

### Axonal RT‐PCR

To collect pure axons, cerebellar GC neurons were grown in microfluidic chambers. Before using TRIzol reagent to dissolve axons, axonal compartments were carefully examined under microscope to see whether any cell soma might migrate into those compartments. 50 *μ*L of TRIzol was applied to each axonal compartment or soma compartment. Lysates from 50 chambers were pooled together, and total RNA was extracted. cDNA was then synthesized using by PrimeScript RT Master Mix. PCR was performed using Taq DNA polymerase (R001B, Takara) with specific primers. The primers used are as following: *β‐actin*: 5’‐ AGGGAAATCGTGCGTGACAT‐3’ and 5’‐ ACGCAGCTCAGTAACAGTCC‐3’, *H1f0*, 5’‐ AGTATATCAAGAGCCACTACAAGG‐3’ and 5’‐AATGTATTTACAGAAAACAGGAGG‐3’. The primers of *Dvl1* and *Wnt5a* were the same as used in real‐time RT‐PCR.

### DiI Labeling

DiI labeling was performed as previously described.^[^ ^25^ ^]^ P0 cerebella from *Ythdf1* and *Ythdf2* cKO mice were fixed in 4% PFA at 4 °C overnight. After washing in PBS, cerebella were embedded in 3% agar and coronally sectioned at 200 µm using a vibratome. Then the lipophilic dye Fast DiI (1 mg mL^−1^ D7756, Thermo) was injected into the EGL of cerebella. After 3 days, sections were mounted and Z‐stack images were taken using confocal microscopy.

### Motor Behavioral Tests

Behavioral tests to test cerebellar functions were conducted as previously described.^[^ ^41^ ^]^ The limb grip strength was measured by a grip strength meter. The mice were put to hold the horizontal grip and pulled backward gradually until they could not hold the grip. Each mouse was tested five times, and the average grip length was recorded and normalized to its body weight. Rotarod was used to evaluate the motor coordination and balance ability of mice. The test was performed for three trials per day on three consecutive days. Each trial was conducted with 10 min intervals to let mice rest in their home cages. Rotarod was set to accelerate from 5 to 40 rpm throughout 300 s. The latency to fall for each mouse was recorded and used in subsequent analysis. Footprint analysis was used to measure gait abnormalities. The hindlimb and forelimb of mice were painted with nontoxic blue and red paints, respectively. Then the mice were let to walk along a strip of white paper under a custom‐made tunnel with 50 cm in length and 10 cm in width. The distance of hindlimb footprints was measured to get the length of stride, sway and stances.

### Statistical Analysis

All experiments were conducted at a minimum of three independent biological replicates in the lab. Data were represented as box and whisker plots with the following settings: 25th–75th percentiles (boxes), minimum and maximum (whiskers), and medians (horizontal lines). Other data are mean ± SEM. Statistical analysis was performed using GraphPad Prism 7.0. When comparing the means of two groups, an unpaired Student's *t* test was performed based on experimental design. When comparing the means of more than two groups, one‐way ANOVA followed by Tukey's multiple comparison test was carried out. A *p*‐value less than 0.05 was considered as statistically significant: *p < 0.05, **p < 0.01, ***p < 0.001, ****p < 0.0001.

## Conflict of Interest

The authors declare no conflict of interest.

## Author Contributions

J.Y., Y.S., and L.Y. contributed equally to this work. S.‐J.J. and J.Y. formulated the idea and designed the experiments. J.Y., Y.S., and L.Y. performed and analyzed most of the experiments. M.Z., P.H., J.L., and X.L. performed some experiments and helped with data analysis. N.W. designed and made the chambers. M.C. made the lentiviral constructs. Y.Z., C.J., and Y.Y. helped with animal work and provided technical help. S.‐J.J. and J.Y. wrote the manuscript.

## Supporting information

Supporting Information

Supporting Information

Supporting Information

Supporting Information

Supporting Information

Supporting Information

Supporting Information

Supporting Information

## Data Availability

The RIP‐seq and YTHDF2‐KD/RNAseq data have been deposited to the Gene Expression Omnibus (GEO) with accession numbers GSE153689 and GSE153688, respectively. The mass spectrometry proteomics data have been deposited to the ProteomeXchange Consortium via the PRIDE partner repository with the dataset identifier PXD019249.
