# Supplementary material for: The m6A Readers YTHDF1 and YTHDF2 Synergistically Control Cerebellar Parallel Fiber Growth by Regulating Local Translation of the Key Wnt5a Signaling Components in Axons
Source: Adv Sci (Weinh). 2021 Oct 12;8(22):2101329. doi: 10.1002/advs.202101329 (PMC8596126; doi:10.1002/advs.202101329)
Supplement: Supplementary file 1 — Supporting Information [file ADVS-8-2101329-s003.pdf]

© 2021 Wiley-VCH GmbH

Supporting Information

for *Adv. Sci.*, DOI: 10.1002/advs.202101329

The m^{6}A Readers YTHDF1 and YTHDF2 Synergistically Control Cerebellar Parallel Fiber Growth by Regulating Local Translation of the Key Wnt5a Signaling Components in Axons

*Jun Yu, Yuanchu She, Lixin Yang, Mengru Zhuang, Peng Han, Jianhui Liu, Xiaoyan Lin, Nijia Wang, Mengxian Chen, Chunxuan Jiang, Yujia Zhang, Yujing Yuan, and Sheng-Jian Ji**

Supporting Information

The m^{6}A Readers YTHDF1 and YTHDF2 Synergistically Control Cerebellar Parallel Fiber Growth by Regulating Local Translation of the Key Wnt5a Signaling Components in Axons

*Jun Yu[#], Yuanchu She[#], Lixin Yang[#], Mengru Zhuang, Peng Han, Jianhui Liu, Xiaoyan Lin, Nijia Wang, Mengxian Chen, Chunxuan Jiang, Yujia Zhang, Yujing Yuan, and Sheng-Jian Ji**

Figures S1~S8

Table S1. List of YTHDF1 target mRNAs by anti YTHDF1 RIP-seq

Table S2. List of YTHDF2 target mRNAs by anti YTHDF2 RIP-seq

Table S3. Proteome analysis after YTHDF1 knockdown

Table S4. Transcriptome analysis after YTHDF2 knockdown

Table S5. Overlapping transcripts of RIP-seq vs Y1-KD_MS or Y2-KD_RNA-seq

Table S6. Transcriptome analysis after YTHDF1 knockdown

Table S7. Overlapping transcripts of Y1-RIP-seq vs Y1-KD_MS or Y1-KD_RNA-seq

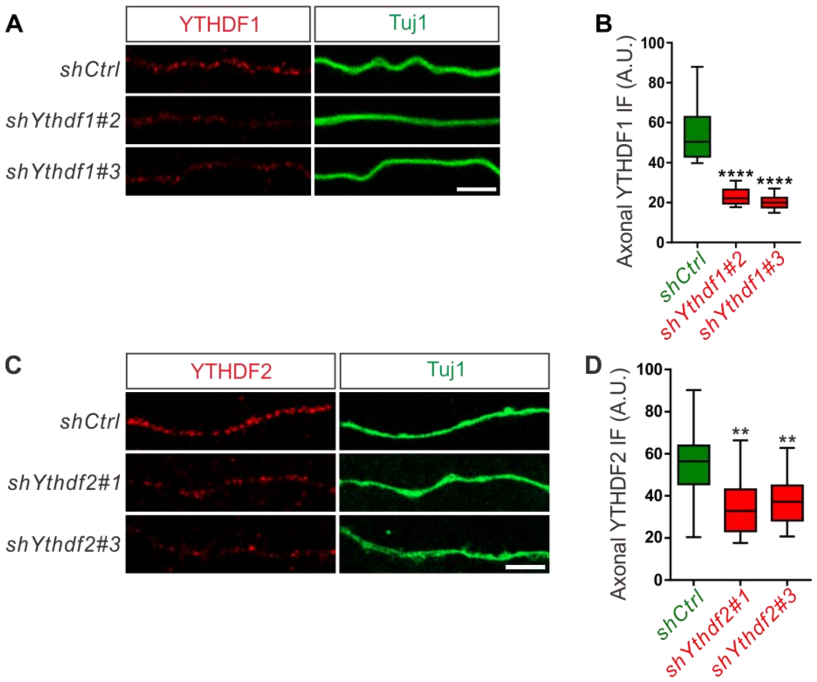

Figure S1. The specificity of YTHDF IF signals in axons were validated by knockdown.

A) Representative confocal images showing YTHDF1 IF signal was significantly reduced in GC axons after *shYthdf1* infection. Tuj1 was used as an axon marker.

B) Quantification of YTHDF1 IF intensity in GC axons (A). Data are represented as box and whisker plots: *shYthdf1#2* (n = 22 axons) vs *shCtrl* (n = 22 axons), ****p = 1.89E-11; *shYthdf1#3* (n = 21 axons) vs *shCtrl*, ****p = 1.89E-06; by one-way ANOVA followed by Tukey's multiple comparison test.

C) Representative confocal images showing YTHDF2 IF was significantly reduced in GC axons after *shYthdf2* infection. Tuj1 was used as an axon marker.

D) Quantification of YTHDF2 intensity in GC axons (C). Data are represented as box and whisker plots: *shYthdf2#1* (n = 15 axons) vs *shCtrl* (n = 15 axons), **p = 0.0029; *shYthdf2#3* (n = 18 axons) vs *shCtrl*, **p = 0.0058; by one-way ANOVA followed by Tukey's multiple comparison test.

Scale bars represent 5 μm (A, C).

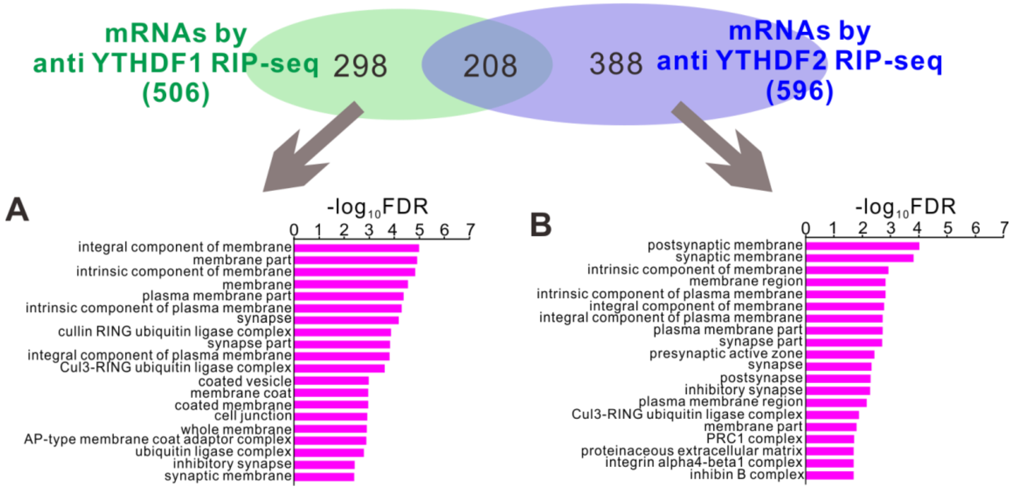

Figure S2. The putative mRNA targets were identified by anti-YTHDF1 and anti-YTHDF2 RIP-seq.

A and B) Gene Ontology (GO) analysis of target mRNAs identified by anti-YTHDF1 (A) and anti-YTHDF2 (B) RIP-seq. The GO terms in Cellular Component are shown.

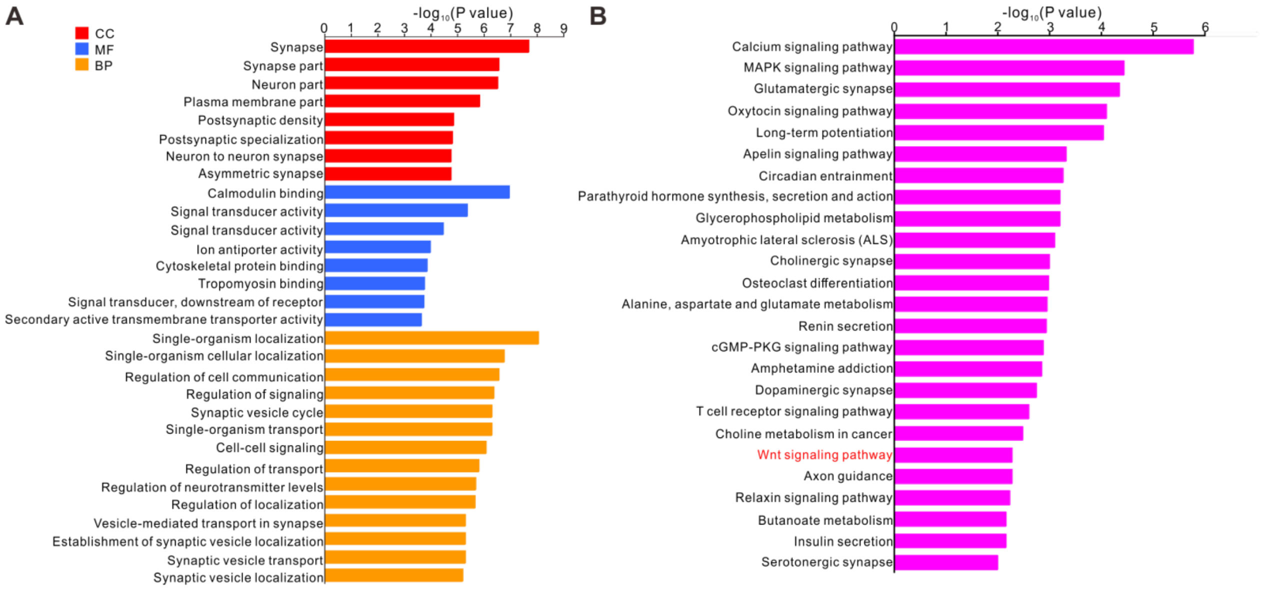

Figure S3. The differentially expressed genes were identified by proteome analysis after YTHDF1 KD.

A) GO analysis of all differentially expressed proteins revealed by quantitative proteomic analysis after YTHDF1 KD in GCs. BP, Biological Process; MF, Molecular Function; CC, Cellular Component.

B) KEGG analysis of all differentially expressed proteins revealed by quantitative proteomic analysis after YTHDF1 KD in GCs. The Wnt signaling pathway is highlighted in red texts.

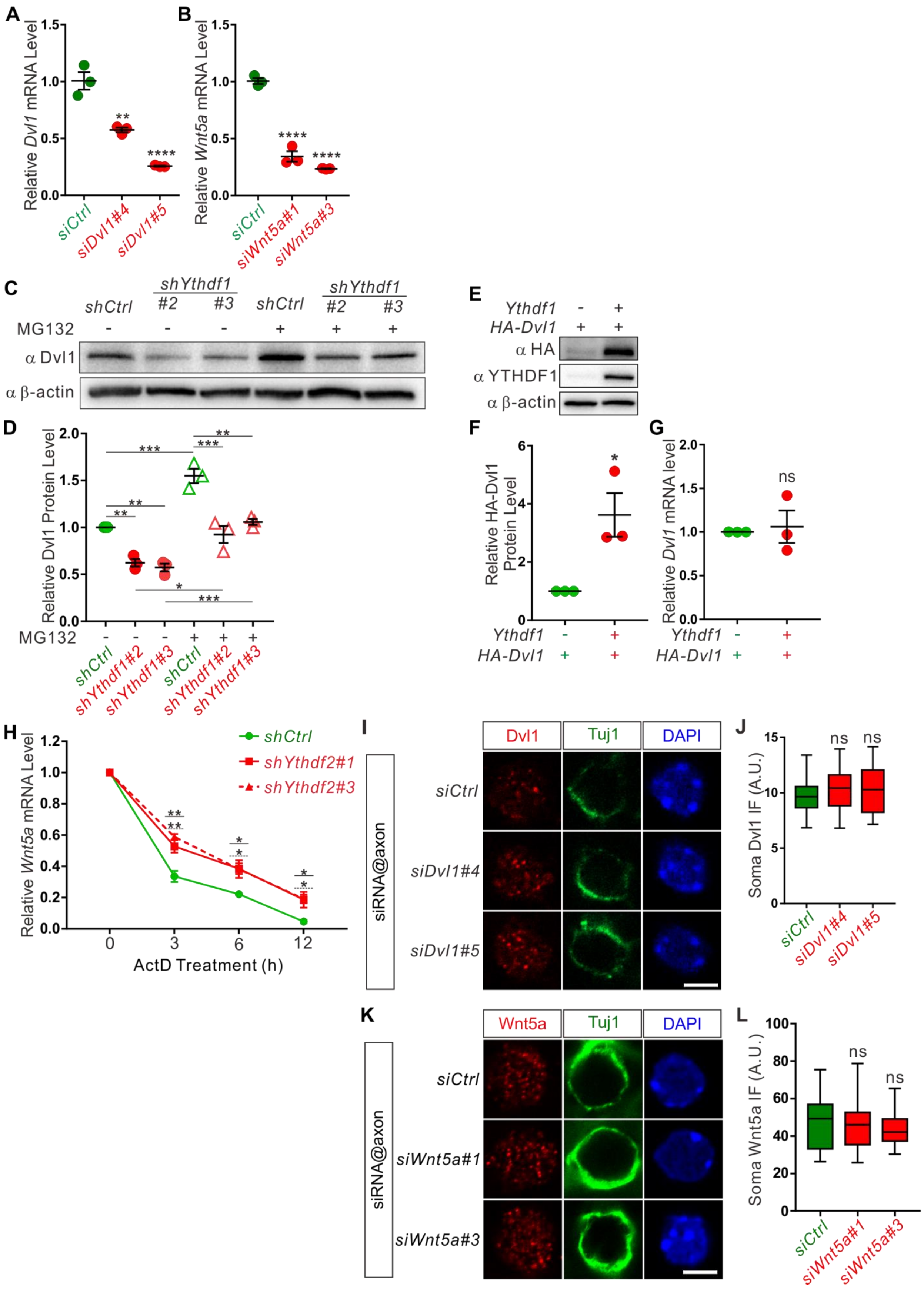

A
Relative Dvl1 mRNA Level
siCtrl
siDvl1#4
siDvl1#5
B
Relative Wnt5a mRNA Level
siCtrl
siWnt5a#1
siWnt5a#3
C
shCtrl
shYthdf1
#2
#3
MG132
α Dvl1
α β-actin
D
Relative Dvl1 Protein Level
shYthdf1#2
shYthdf1#3
E
Ythdf1
HA-Dvl1
α HA
α YTHDF1
F
Relative HA-Dvl1 Protein Level
G
Relative Dvl1 mRNA level
ns
H
Relative Wnt5a mRNA Level
shYthdf2#1
shYthdf2#3
ActD Treatment (h)
I
Dvl1
Tuj1
DAPI
siRNA@axon
J
Soma Dvl1 IF (A.U.)
K
Wnt5a
L
Soma Wnt5a IF (A.U.)

Figure S4. YTHDF1 and YTHDF2 regulate translation of *Dvl1* and stability of *Wnt5a*, respectively.

A and B) Knockdown efficiencies of siRNAs against *Dvl1* (A) and *Wnt5a* (B) confirmed by RT-qPCR. Data are mean ± SEM. For A, *siDvl1#4* vs *siCtrl*, **p = 0.0014; *siDvl1#5* vs *siCtrl*, ****p = 6.60E-05. For B, *siWnt5a1#1* vs *siCtrl*, ****p = 1.10E-05; *siWnt5a1#3* vs *siCtrl*, ****p = 4.28E-06. n = 3 replicates, all by one-way ANOVA followed by Tukey's multiple comparison test.

C and D) WB confirming the Dvl1 protein level decreased after knockdown of YTHDF1 in GCs and this downregulation was not affected by MG132 treatment. Quantification data are mean ± SEM (D). *shYthdf1#2* vs *shCtrl*, **p = 0.0048; *shYthdf1#3* vs *shCtrl*, **p = 0.0017; MG132+*shCtrl* vs *shCtrl*, ***p = 1.78E-04; MG132+*shYthdf1#2* vs MG132+*shCtrl*, ****p = 4.95E-05; MG132+*shYthdf1#3* vs MG132+*shCtrl*, ***p = 5.10E-04; MG132+*shYthdf1#2* vs *shYthdf1#2*, *p = 0.024; MG132+*shYthdf1#3* vs *shYthdf1#3*, ***p = 5.68E-04; n = 3; by one-way ANOVA followed by Tukey's multiple comparison test.

E-G) WB showing that co-transfection of *Ythdf1* and *HA-Dvl1* expression plasmids in HEK293T cells increased Dvl1 protein level without changing *Dvl1* mRNA level. Quantification data are mean ± SEM (F and G). For F, *p = 0.025; for G, p = 0.77; ns, not significant; by unpaired Student's t test.

H) Knockdown of YTHDF2 increased stability of the *Wnt5a* mRNA in GCs. Actinomycin D (ActD) was added to GCs infected with lenti viral shRNAs to inhibit transcription. Neurons were collected at different time points and RNAs were purified. RT-qPCR confirmed that stability of the *Wnt5a* mRNA level was increased after knockdown of YTHDF2 in GCs. Data are mean ± SEM. For 3h, *shYthdf2#1* vs *shCtrl*, **p = 0.0062; *shYthdf2#3* vs *shCtrl*, **p = 0.0010. For 6h, *shYthdf2#1* vs *shCtrl*, *p = 0.035; *shYthdf2#3* vs *shCtrl*, *p = 0.038. For 12h, *shYthdf2#1* vs *shCtrl*, **p = 0.040; *shYthdf2#3* vs *shCtrl*, *p = 0.034. n = 4; by unpaired student's t test.

I and J) Compartmentalized knockdown of Dvl1 in axons did not affect Dvl1 protein level in GC soma. Quantification data are represented as box and whisker plots (J). *siDvl1#4* (n = 28 cells) vs *siCtrl* (n = 25 cells), p = 0.54; *siDvl1#5* (n = 25 cells) vs *siCtrl*, p = 0.53; ns, not significant; by one-way ANOVA followed by Tukey's multiple comparison test.

K and L) Compartmentalized knockdown of Wnt5a in axons did not affect Wnt5a protein level in GC soma. Quantification data are represented as box and whisker plots (L). *siWnt5a#1* (n = 29 cells) vs *siCtrl* (n = 28 cells), p = 0.78; *siWnt5a#3* (n = 29 cells) vs *siCtrl*, p = 0.51; ns, not significant; by one-way ANOVA followed by Tukey's multiple comparison test.

Scale bars represent 5 μm (I, K).

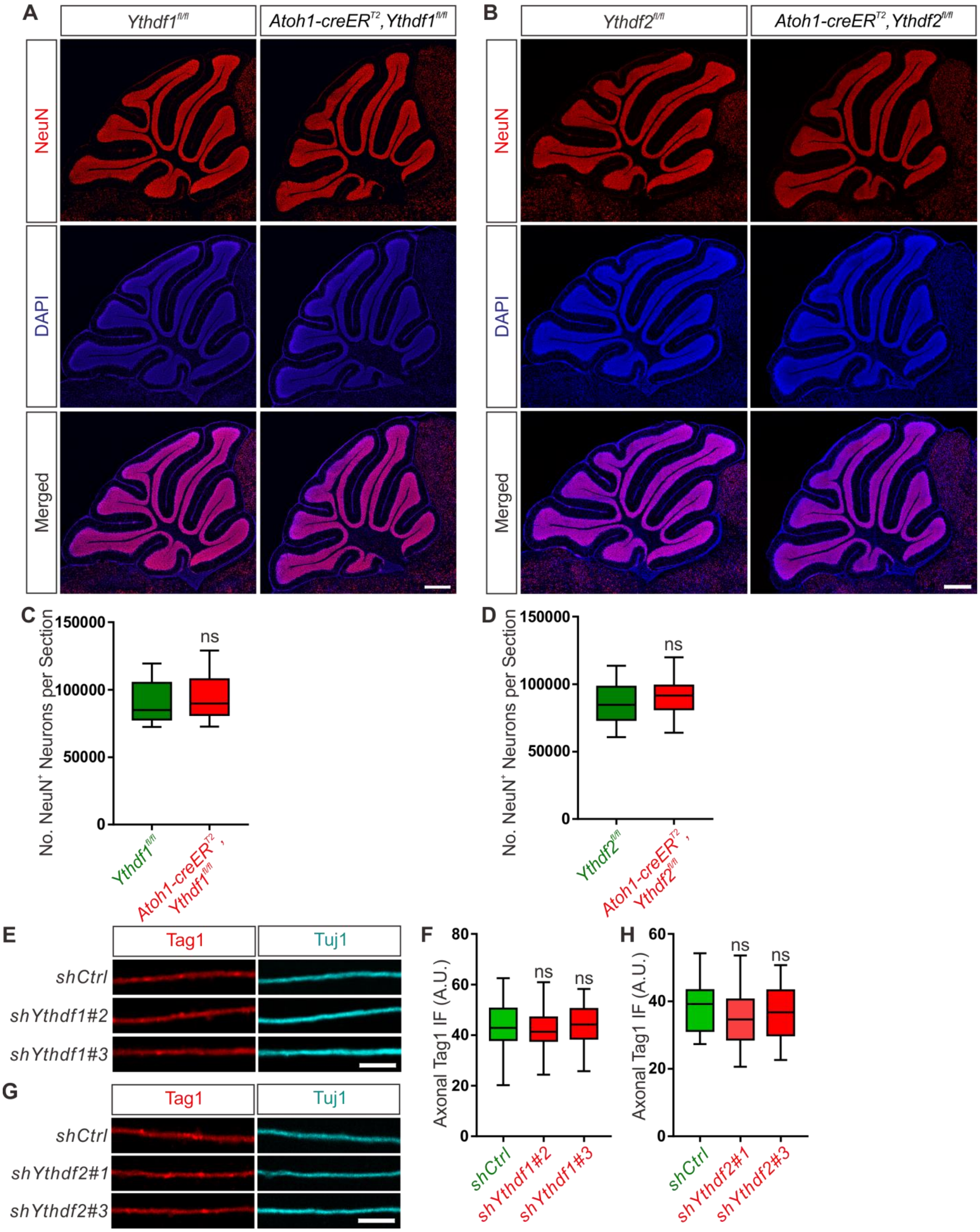

Figure S5. Conditional knockout of *Ythdf1* or *Ythdf2* does not affect neurogenesis of GCs.

A-D) Representative images of NeuN immunostaining in P15 cerebellum of *Ythdf1* (A) and *Ythdf2* (B) cKO mice. The number or pattern of mature GCs showed no change in cKO mice compared with control littermates. The numbers of $NeuN^+$ GC neurons per section were quantified (C and D). Data are expressed as box and whisker plots. For C, $Ythdf1^{fl/fl}$ (n = 12 confocal fields) vs *Ythdf1* cKO (n = 11 confocal fields), p = 0.78; for D, $Ythdf2^{fl/fl}$ (n = 10 confocal fields) vs *Ythdf2* cKO (n = 10 confocal fields), p = 0.52; ns, not significant; by unpaired Student's t test. Scale bars represent 500 μm (A and B).
E-H) Knockdown of YTHDF1 or YTHDF2 did not change axonal Tag1 protein level in GCs. Quantification of axonal Tag1 IF in E and G was shown in F and H, respectively. Data are represented as box and whisker plots. For F, p = 0.79 (*shYthdf1#2* vs *shCtrl*), p = 0.98 (*shYthdf1#3* vs *shCtrl*); for H, p = 0.31 (*shYthdf2#1* vs *shCtrl*), p = 0.64 (*shYthdf2#3* vs *shCtrl*); ns, not significant; by one-way ANOVA followed by Tukey's multiple comparison test. Scale bars represent 5 μm (E and G).

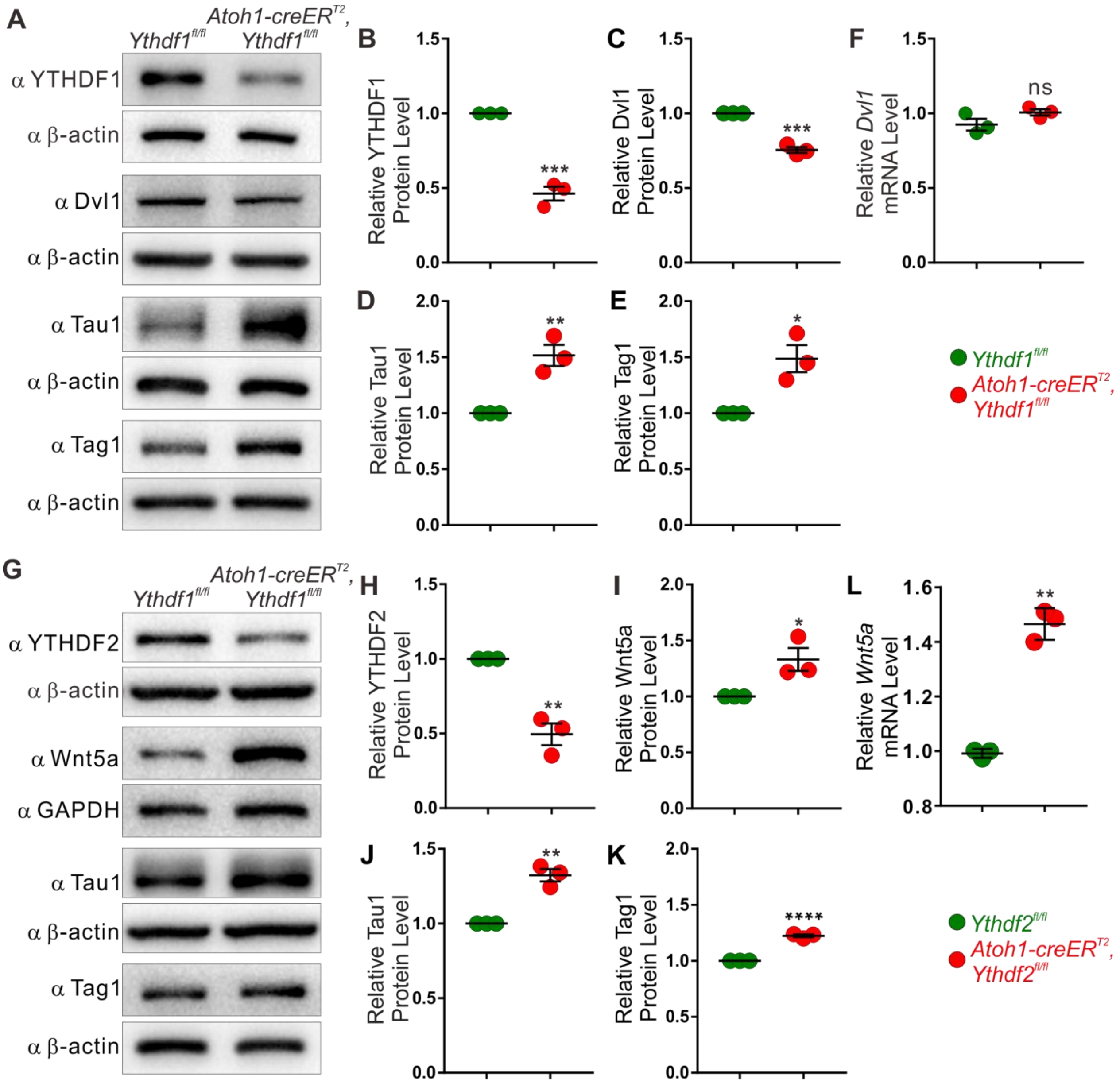

Figure S6. YTHDF1 and YTHDF2 regulate translation of *Dvl1* and stability of *Wnt5a* respectively *in vivo* to promote axon growth.

A-E) Representative immunoblots showing that Dvl1 level was reduced and protein levels of axon markers were increased in P15 *Ythdf1* cKO cerebellum (A). Quantification of WB for YTHDF1 (B), Dvl1 (C), Tau1 (D) and Tag1 (E). For B, ***p = 0.00031; for C, ***p = 0.00023; for D, **p = 0.0054; for E, *p = 0.016; n = 3 replicates; by unpaired Student' t test.

F) *Dvl1* mRNA level is not changed in the cerebellum of *Ythdf1* cKO mice. Data are expressed as dot plots. p = 0.14; ns, not significant; by unpaired Student's t test.

G-K) Representative immunoblots showing that Wnt5a level was upregulated and protein levels of axon markers were increased in P15 *Ythdf2* cKO cerebellum (G). Quantification of WB for YTHDF2 (H), Wnt5a (I), Tau1 (J) and Tag1 (K). For H, **p = 0.0023; for I, *p = 0.032; for J, **p = 0.0014; for K, ****p = 4.56E-05; n = 3 replicates; by unpaired Student' t test.

L) *Wnt5a* mRNA level is significantly increased in the cerebellum of *Ythdf2* cKO mice. Data are expressed as dot plots. **p = 0.0026; by unpaired Student's t test.

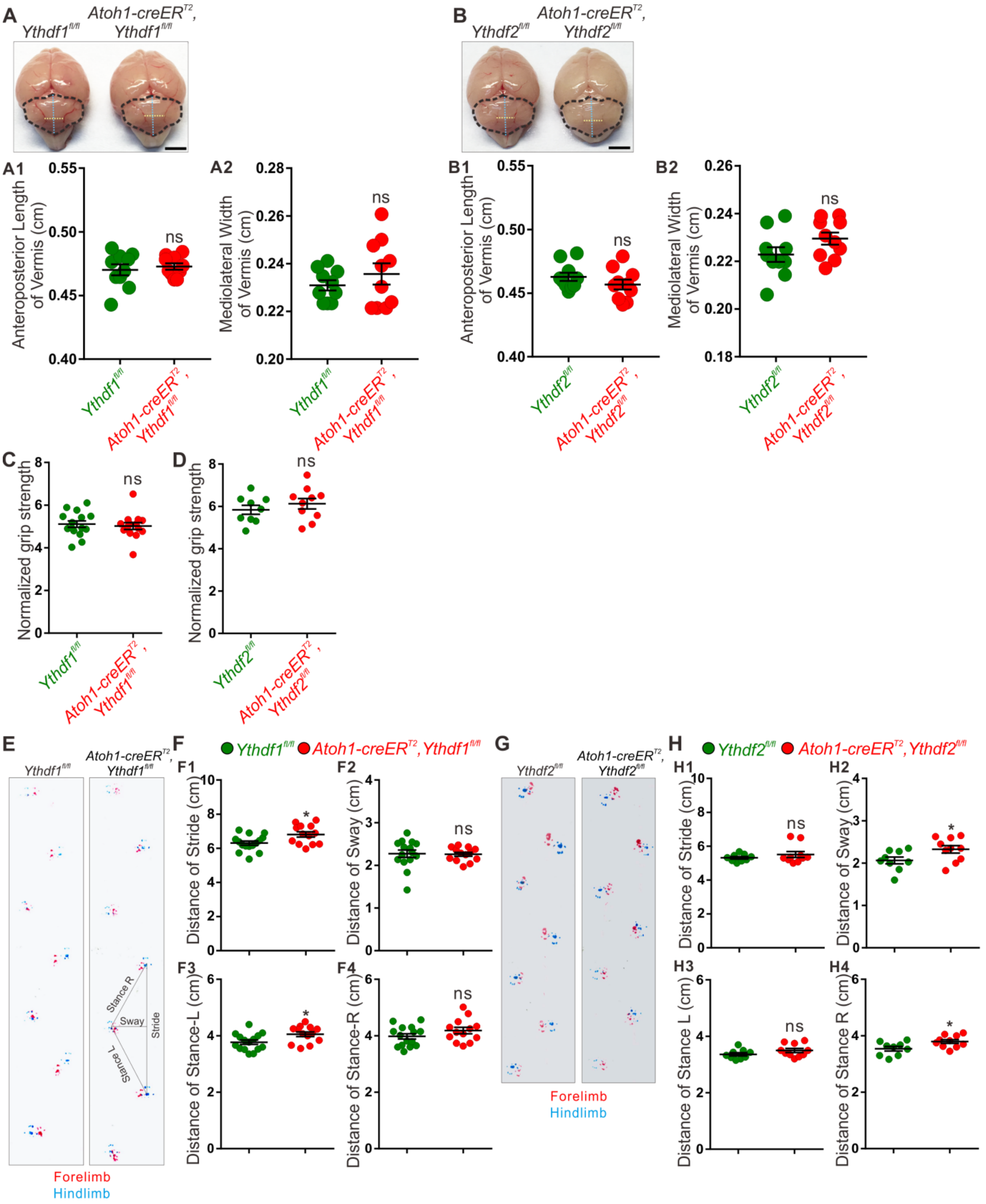

Figure S7. Grip Strength and Footprint Tests Were Carried Out for *Ythdf1* and *Ythdf2* cKO Mice.

A and B) The sizes of *Ythdf1* (A) and *Ythdf2* (B) cKO cerebella were quantified by measuring the anteroposterior length (the blue dotted lines) and the mediolateral width (the yellow dotted lines) of the

vermis. Quantification data are shown in A1, A2, B1 and B2: A1, p = 0.65; A2, p = 0.35; B1, p = 0.24; B2, p = 0.11; n = 10 mice for each genotype; ns, not significant; by unpaired Student's t test.

C and D) Measurements of normalized forelimb grip strength showing no difference for *Ythdf1* (A) and *Ythdf2* (B) cKO with their control mice. In A, p = 0.69; n = 15 for $Ythdf1^{fl/fl}$ mice; n = 14 for *Ythdf1* cKO mice. In B, p = 0.39; n = 9 for $Ythdf2^{fl/fl}$ mice; n = 10 for *Ythdf2* cKO mice. ns, not significant; by unpaired Student's t test.

E) Representative images of footprints (red, forelimb; blue, hindlimb) of $Ythdf1^{fl/fl}$ and *Ythdf1* cKO mice.

F) Quantification of the distance of stride (F1), sway (F2), stance-L (F3) and stance-R (F4). For stride, *p = 0.015; for sway, p = 0.89; for stance-L, *p = 0.019; for stance-R, p = 0.17; n = 15 for $Ythdf1^{fl/fl}$ mice; n = 14 for *Ythdf1* cKO mice; ns, not significant; by unpaired Student's t test.

G) Representative images of footprints (red, forelimb; blue, hindlimb) of $Ythdf2^{fl/fl}$ and *Ythdf2* cKO mice.

H) Quantification of the distance of stride (H1), sway (H2), stance-L (H3) and stance-R (H4). For stride, p = 0.34; for sway, *p = 0.043; for stance-L, p = 0.17; for stance-R, *p = 0.024; n = 9 for $Ythdf2^{fl/fl}$ mice; n = 10 for *Ythdf2* cKO mice; ns, not significant; by unpaired Student's t test.

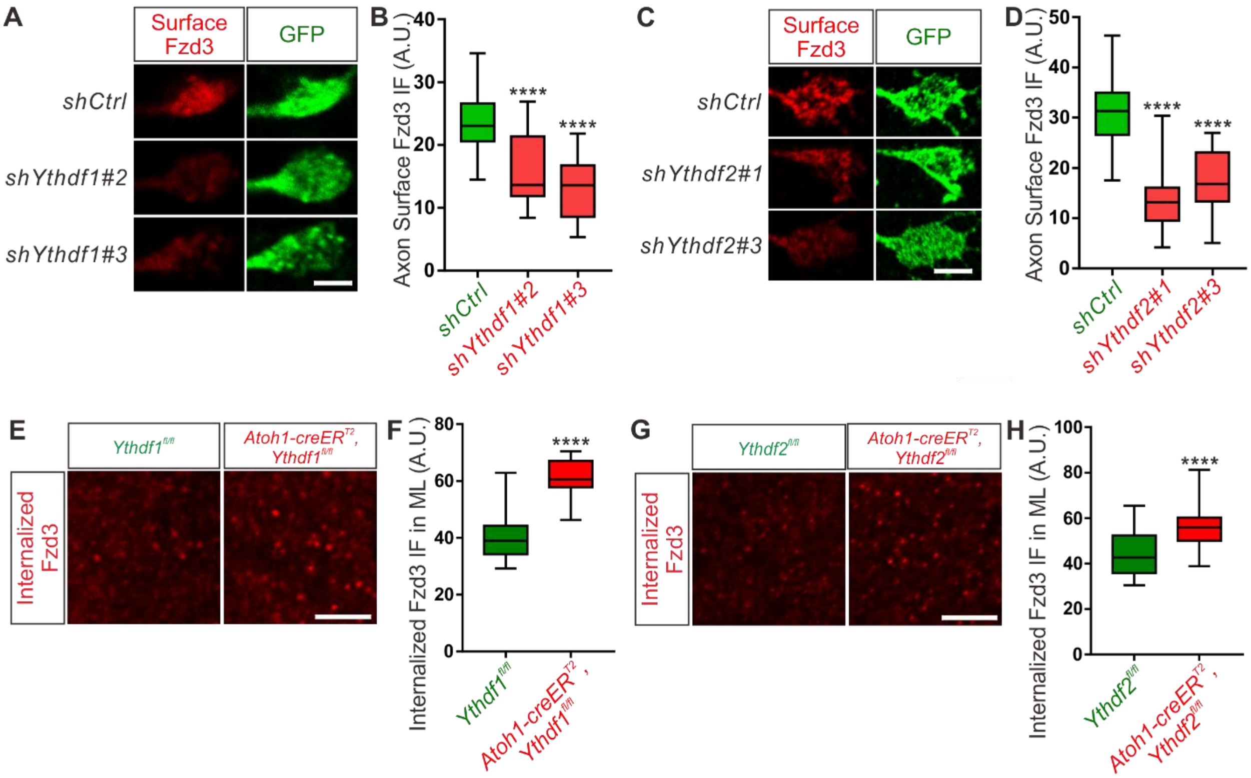

Figure S8. Loss-of-function of YTHDF1 or YTHDF2 induced internalization of Frizzed3 in GC axons.

A-D) Knockdown of YTHDF1 or YTHDF2 induced internalization of Frizzed3 in cultured GC axons. Representative confocal images of Frizzed3 (Fzd3) IF on the membrane surface of GC growth cones after YTHDF1 (A) or YTHDF2 (C) KD without permeabilization were shown. Quantification of IF was shown in B and D. Data are represented as box and whisker plots: in B, ****p = 2.48E-05 (*shYthdf1#2* vs *shCtrl*), ****p = 2.38E-08 (*shYthdf1#3* vs *shCtrl*); in D, ****p = 1.59E-11 (*shYthdf2#1* vs *shCtrl*), ****p = 1.58E-08 (*shYthdf2#3* vs *shCtrl*); n = 20 axons for each condition; by one-way ANOVA followed by Tukey's multiple comparison test. Scale bars represent 5 μm (A, C).

E-H) Increases of internalized Frizzed3 were detected in *Ythdf1* and *Ythdf2* cKO mice. P15 cerebellar sections were prepared with cryostat and permeabilized with Triton x-100 to expose the internalized Fzd3 protein. Representative confocal images of internalized Frizzed3 (Fzd3) IF in molecular layer of cerebella were shown for *Ythdf1* (E) and *Ythdf2* (G) cKO mice. Quantification of IF was shown in F and H. Data are represented as box and whisker plots: in F, ****p = 1.04E-13, n = 25 confocal fields; in H, ****p = 4.58E-8, n = 40 confocal fields; by unpaired Student's t test. Scale bars represent 5 μm (E, G).
